# Educational intervention for nursing professionals on palliative care: quasi-experimental study*

**DOI:** 10.1590/1518-8345.8026.4822

**Published:** 2026-06-15

**Authors:** Joyce Assunção Barros, Taciana Fernandes Araújo Ferreira, Fabiana Bolela, Raquel Pan, Silmara Elaine Malaguti Toffano, Adriana Cristina Nicolussi

**Affiliations:** 1 Universidade Federal do Triângulo Mineiro, Uberaba, MG, Brazil.; 2 Scholarship holder at the Fundação de Amparo à Pesquisa do Estado de Minas Gerais (FAPEMIG), Brazil.; 3 Universidade de São Paulo, Escola de Enfermagem de Ribeirão Preto, PAHO/WHO Collaborating Centre for Nursing Research Development, Ribeirão Preto, SP, Brazil.

**Keywords:** Palliative Care, Nursing Team, Continuing Nursing Education, Knowledge, Self Efficacy, Tertiary Healthcare

## Abstract

**Objective:**

to assess nursing professionals› knowledge and self-efficacy regarding palliative care before and after an educational intervention.

**Method:**

a quasi-experimental study conducted in a teaching hospital with nursing professionals who care for adults and the elderly. Data collection was performed at two times: first, a sociodemographic questionnaire and the Bonn Palliative Care Knowledge Test, which assesses knowledge and self-efficacy in palliative care, were administered; immediately afterward, an educational intervention on the topic was conducted. Four weeks later, the instrument that assesses knowledge and self-efficacy was reapplied. The data were analyzed using the Statistical Package for the Social Sciences (SPSS) version 21.0.

**Results:**

179 professionals participated, with an average age of 40.17 years and a predominance of females. The knowledge score obtained higher averages after the intervention (pre-test=11.77; post-test=15.03), while the self-efficacy score was lower (pre-test=3.21; post-test=3.38), but both were statistically significant (p<0.01).

**Conclusion:**

the educational intervention was effective in increasing knowledge and self-efficacy in palliative care among nursing professionals. These results may support the development and dissemination of palliative care practices in health services.

## Highlights

**(1)** Educational intervention had a positive impact on the professionals’ knowledge. **(2)** The number of correct answers after the educational intervention increased. **(3)** The teaching-learning process in palliative care should be encouraged. **(4)** The results can support the development of other educational interventions. **(5)** The implementation of this care can provide higher-quality assistance.

## Introduction

Palliative care (PC) is intended for people with life-threatening illnesses or conditions that cause limitations and progressive loss of functionality. Its main benefits and objectives are to prevent and treat the suffering that patients, families and caregivers may experience, whether physical, psychological, social and/or spiritual^1^.

Despite many advances in Brazil, PC is still not widely available, both in specialized services and around education, given that there are gaps in the knowledge of health professionals, a lack of training for existing teams and an absence of content in the undergraduate curriculum^2^.

An analysis of healthcare professionals’ understanding of PC showed that they have little knowledge or a distorted view of the subject. These findings are concerning, as a lack of training leads to uncertainty about how to act in specific situations, depriving patients and their families of the benefits that PC can provide^3^.

The Brazilian Ministry of Health (BMoH) has been contributing to the implementation of this approach in the country. On December 7, 2023, Resolution No. 729 was published, approving the National Palliative Care Policy (PNCP, acronym in Portuguese) in the Brazilian Unified Health System (SUS, acronym in Portuguese), which guarantees access to this care at any point in the Health Care Network (RAS, acronym in Portuguese). Given this scenario, it is understood that the demand related to the care of these patients will not be met solely by teams specialized in PC, as there are insufficient numbers of such teams in Brazil. Thus, non-specialist professionals already working in the RAS would be responsible for meeting most of the demands related to PC^4^.

However, for this to happen, these professionals need to develop knowledge and skills in this subject so they can apply the practice correctly, following the principles and providing quality care without leaving the population unattended. Nursing professionals are responsible for meeting most of the demands; therefore, educational actions are necessary as part of health system planning to favor not only access but also the resolution of the health needs of users who require this care^5^.

In-service education is a continuous and dynamic process for building professional knowledge. Educational activities promote critical thinking and transform the reality in which professionals operate. In addition, nursing practices are always associated with educational actions that promote changes in their activities, ensure the quality of care, and foster continuous professional improvement^6^.

Thus, given the low level of development of PC in Brazil, possibly associated with gaps in professionals’ knowledge and considering that it is an essential practice in health systems, especially in this context of sociodemographic transition, with a growing demand from patients who can benefit from this approach, it is necessary to develop actions aimed at building knowledge and raising awareness on the subject, with a view to improving the care provided to the population^5^.

In this context, identifying what knowledge professionals have should be considered in teaching practice, as it facilitates the learning process and its transformation into practical actions to change reality^7^. Given the above scenario, this study aimed to assess nursing professionals’ knowledge and self-efficacy regarding PC before and after an educational intervention.

## Method

### Study design

This is a quasi-experimental, before-and-after study in which the same subjects were evaluated at pre-test and post-test points during an educational intervention that followed the Template for Intervention Description and Replication (TIDieR).

### Location

The study was conducted at a public teaching hospital in Uberaba, in the state of Minas Gerais (MG), Brazil. The hospital has 302 active beds and serves the demand of the SUS for the Minas Gerais macro-region. The institution is a reference for medium- and high-complexity cases^8^.

### Period

Data collection was carried out from May to September 2024.

### Sample and inclusion criteria

The study population consisted of nursing professionals (nurses, nursing technicians, and nursing assistants) aged 18 years or older who worked in direct care for adult/elderly patients in the following inpatient sectors: orthopedics, internal medicine, surgical clinics, infectious and parasitic diseases unit, neurology, gynecology and obstetrics, onco-hematology, intensive care unit I, II and coronary care unit and adult emergency room. Professionals who were on vacation or away from work during the study period were excluded.

The sample size was calculated based on the pilot study, which included 18 professionals. The significance level considered was a type I error (α=0.01), a type II error (β=0.1) and a statistical power of 90%. Considering knowledge of PC as the primary outcome, the mean increase in scores from pre-test to post-test was 5.72 points (Sd=3.10), equivalent to a gain of more than 50%. Based on these values, the sample size was calculated using the Power Analysis and Sample Size (PASS) program, resulting in a total of 30 participants. It should be noted that the participants in the pilot study were not part of the main study.

Given that the magnitude of the intervention effect in the pilot study was greater than expected, it was decided, as a precaution, that the main study should include a larger number of participants than the pilot study recommended. As the intervention was carried out in partnership and registered with the hospital’s Nursing Education Service (SEE, in Portuguese) above, it was decided to invite all nursing professionals interested in participating in the study. Therefore, a non-probabilistic convenience sampling was used.

Thus, 214 nursing professionals participated in the pretest; however, during the post-test application stage, 35 participants were lost, resulting in a final sample of 179.

### Data collection instruments

A sociodemographic and professional questionnaire developed by the authors themselves was used to characterize the sample, containing the following variables: age, sex, level of education, professional category, academic degree, specialization (postgraduate), sector of employment, total professional experience and work shift. 

To evaluate knowledge and self-efficacy in PC, the Bonn Palliative Care Knowledge Test (BPW) was used, developed in Germany in 2011^9^ and translated and culturally adapted to the Brazilian context in 2018^10^. It contains 38 questions, 23 of which are intended to assess knowledge, covering pain and other symptom control, general understanding of the subject and attitudes toward death and dying. According to the instrument’s instructions, knowledge items numbered 1 to 4, 6 to 10, 12, 14 and 16 to 20 should be considered incorrect, while items 5, 11, 13, 15 and 21 to 23 are considered correct.

The remaining 15 questions assess self-efficacy in providing PC and gauge the participant’s confidence in applying what they have learned in clinical practice. The questions are organized in a Likert scale format, with responses including “correct”, “more correct than incorrect”, “more incorrect than correct” or “incorrect”^9^ ^-^ ^10^.

On the knowledge scale, the overall score is the sum of the items the respondent answered correctly, ranging from 0 (if the respondent got all items wrong) to 23 (if they got all items right). The higher the score, the greater the professional’s knowledge. On the self-efficacy scale, the overall score represents the average of the responses to the items on the scale and can range from zero (feels incapable) to four (feels totally capable), which means the closer to four, the more capable the professional feels to perform a specific action described in the instrument^9^ ^-^ ^10^.

### Operational stage

The program content of the educational intervention class was developed based on the PC manual of the Ministry of Health in partnership with the *Sírio-Libanês* Hospital (2023)^1^ and current literature, focusing on essential issues for PC knowledge.

Thus, the topics selected and elaborated were: a) updated concept and principles of PC; b) the importance of the topic for health professionals, with presentation of epidemiological data; c) the relationship between PC and disease-modifying treatment, highlighting that the objectives of palliative therapy will be in accordance with the functional and clinical evolution of each patient; d) the importance of multidisciplinary care; e) identification and control of physical symptoms prevalent in this demand (pain, nausea and vomiting, constipation and dyspnea); f) the main psychological symptoms (anxiety, depression and *delirium*); g) the most common social aspects, such as loss of social identity, loss of social and family role and social isolation; h) spirituality; i) end-of-life care, emphasizing that the proposed treatments should be evaluated in relation to their proportionality considering the process of finitude; j) effective communication between the team, patient and family; k) the services that are available at the hospital to meet the demand for PC and how to request this service.

After preparing the lesson, the content was sent for validation, which was carried out independently via an invitation letter sent electronically (via email) to 13 doctors previously selected through analysis of their Lattes Curricula, who knew the construct in question. Of the 13 doctors, seven did not respond to any of the electronic contacts and three refused to participate. Thus, three doctors took part in the content validation.

For those who agreed to participate in the validation, a new email was sent containing the Informed Consent Form (ICF) and the Health Education Content Validation Instrument (IVCES, acronym in Portuguese). The deadline requested for the specialist to return the instrument to the researchers was 10 days, which could be extended if necessary.

The IVCES has 18 items, divided into three groups: objectives (1-5), structure and presentation (6-15) and relevance (16-18), with response options on a Likert scale: 0 = disagree, 1 = partially agree and 2 = totally agree. In addition to the instrument score, the specialist could also record their considerations in continuous text if necessary^11^.

After all specialists had completed and returned the instrument, the judges’ agreement regarding content validation was assessed. To this end, the Content Validity Index (CVI) was calculated as the ratio of responses in agreement (items marked as 2) to the total number of responses, with a minimum acceptable value of 80% considered acceptable agreement. 

Expert 1 awarded the maximum score (100%), selecting “strongly agree” for all 18 items in the instrument. Experts 2 and 3 scored 80%, with 15 items rated “strongly agree” and 3 “partially agree”.

It should be noted that this validation process is essential because it assesses the clarity and understanding of the target audience, its representativeness in adequately addressing the universe it proposes to address, and seeks to avoid the inclusion of unnecessary elements^11^.

After validation, the educational intervention was registered in the hospital’s SEE, as shown above. Meetings were held with the researchers, the SEE nurse and the Technical Managers (TM) of each participating sector to define and schedule the classes’ dates and times. The TM were responsible for disseminating information and directing the nursing team to participate in the intervention.

Next, the educational intervention was tested in a pilot study with 18 professionals and adjustments were made only to the presentation structure, without changing the content, to optimize the intervention time within the scheduled time. After the pilot study, the main study’s educational intervention phase was initiated.

### Data collection

The educational intervention was delivered through an expository presentation supported by Microsoft PowerPoint^®^ visual aids. It also used a participatory and dialogical methodology, aiming to facilitate the exchange of experiences, clarify doubts and discuss the issues raised by participants about the topic.

Data collection was carried out in two stages (pre-test and post-test). The first stage was conducted collectively and in small groups with the nursing teams in a classroom at the hospital, with dates and times scheduled in advance during the professionals’ working hours, across three shifts (morning, afternoon, and night). Several class times and days were necessary because professionals could not leave at the same time from each sector, resulting in a total of 51 classes.

The research objectives were presented at each meeting and ICF was read. After acceptance and signature, the sociodemographic and professional questionnaire and the BPW instrument were applied. An approximate time of 20 minutes was set to complete the instruments, which were completed individually. This time was in accordance with the authors’ recommendations for the original study of the instrument and was also determined based on the pilot study. Next, the researchers themselves conducted in-service health education on PC. The class lasted approximately forty minutes, totaling about one hour for the entire activity.

In the second stage of data collection, four weeks after the educational intervention, only the BPW instrument was reapplied. This stage was conducted individually, through an active search for participants in the sectors in which they worked. Completing the instrument took approximately ten minutes.

### Data processing and analysis

The collected data were entered into an Excel^®^ spreadsheet in Windows^®^ using the double-entry technique for subsequent validation. A code identified each study participant. The validated database was then imported into Statistical Package for the Social Sciences (SPSS) version 21.0 for processing and analysis^12^.

The data were analyzed using descriptive statistics, including measures of central tendency (mean) and dispersion (standard deviation) for quantitative variables and absolute and relative frequencies for categorical variables. The difference in means between the pre-test and post-test was evaluated for both knowledge and self-efficacy in PC. All requirements for the use of parametric tests were duly verified, including data normality and outlier analysis. Thus, the paired T-test was applied, with a significance level of α=0.01 and considering a 99% confidence interval.

To verify the magnitude of the intervention’s effect, an application available on the Psychometrica.de website (https://www.psychometrica.de/effect_size.html)^13^ was used. The interpretation of effect sizes followed Cohen’s classification: Cohen’s d (0.0 to 0.1) = no effect; Cohen’s d (0.2 to 0.4) = small effect; Cohen’s d (0.5 to 0.7) = moderate effect and Cohen’s d (0.8 to ≥1.0) = large effect.

### Ethical aspects

The research was registered in the *Rede Pesquisa* (Research Network), a tool of the Brazilian Hospital Services Company Network (EBSERH, in Portuguese), under code no. 3738 and obtained the consent of the Teaching and Research Management of the hospital mentioned above and was approved by the Research Ethics Committee of the proposing institution, opinion no. 6,510,776, respecting ethical principles.

## Results

Of the 179 participants evaluated in this study, the majority (82.1%) were female, with a mean age of 40.17 years (Sd = 8.5) and a range of 23 to 64 years.

Regarding professional data, the majority (69.3%) were nursing technicians, predominantly working in the medical clinic sector (26.3%) and night shifts (36.3%), with more than 10 years of professional experience (64.2%). Regarding postgraduate education, 44.1% reported having taken specialization courses, predominantly in intensive care (30.37%) and urgent and emergency care (26.58%). Only one participant reported having a postgraduate degree in PC.

Regarding PC knowledge and self-efficacy scores, the number of correct answers increased after the educational intervention, as shown in Table 1.

**Table 1:** Distribution of sample scores measured by the Bonn Palliative Care Knowledge Test (n = 179). Uberaba, MG, Brazil, 2024

Variables	Mean	Sd*	Minimum	Maximum
Pre-test knowledge score	11.77	2.56	4	18
Post-test knowledge score	15.03	3.64	8	23
Pre-test self-efficacy score	3.21	0.48	1.73	4
Post-test self-efficacy score	3.38	0.43	2	4

*Sd = Standard deviation

As for knowledge constructs, the averages increased considerably after in-service training, with a 3-point gain in average performance. The results for self-efficacy also increased, though to a lesser extent, with a gain of only 0.1. Both were considered statistically significant (p<0.01). Regarding the effect of the intervention’s magnitude, the knowledge test was considered significant (0.8 to 1.0) and the self-efficacy test had a moderate impact (0.5 to 0.7), as shown in Table 2. It is worth noting that, for the instrument assessments, the closer to 23, the greater the knowledge and the closer to four, the greater the perceived self-efficacy.

**Table 2:** Comparison of the difference in means between the pre-test and post-test of the nursing team of the Bonn Palliative Care Knowledge Test (n = 179). Uberaba, MG, Brazil, 2024

	Paired samples test			
	Mean	Sd*	*p* ^†^	d^‡^
Pre- and post-test knowledge score	-3.26	4.10	0.000	0.83
Pre- and post-test self-efficacy score	-0.17	0.49	0.000	0.52

*Sd = Standard deviation; ^†^P-value = Significant <0.001; ^‡^d = Cohen’s d

Table 3 presents the pre-test and post-test results for each item of the BPW knowledge assessment and Table 4 presents the self-efficacy assessment results. The question that showed the most significant increase in correct answers after the intervention was item two (percentage difference of 35.2%), which refers to the use of anti-inflammatory drugs with opioids. In the data on self-efficacy, there was less variance between the pre-test and post-test, since the scale ranged only from 1 to 4. It should also be noted that the pre-test results were close to the limit value. Among the items that showed the most significant gain after in-service training, item 26 stood out (percentage difference of 17.2%), which addresses information about CP at the institution itself.

**Table 3:** Evaluation of each pre-test and post-test knowledge item by the nursing staff of the Bonn Palliative Care Knowledge Test (n = 179). Uberaba, MG, Brazil, 2024

Questions	Correct answers		
	Pós-teste	Post-test	
Part 1 - Knowledge	n (%)*	n (%)*	Diff (%)^†^
1. Palliative care should never be combined with curative treatments.	120 (67.0)	161 (89.9)	22.9
2. Non-steroidal anti-inflammatory drugs should not be used during regular opioid use.	91 (50.8)	154 (86.0)	35.2
3. Subcutaneous fluid administration (hypodermoclysis) is necessary to relieve dry mouth (xerostomia) in end-of-life patients.	97 (54.2)	132 (73.7)	19.7
4. The use of transdermal patches to control unstable pain is suitable for patients at the end of life.	55 (30.7)	102 (57.0)	26.3
5. Adjuvant therapies (e.g., physiotherapy) are essential for pain control.	142 (79.3)	156 (87.2)	7.9
6. For family members, it is always essential to remain by the side of the dying patient.	13 (7.3)	48 (26.8)	18.9
7. Constipation should be accepted as a secondary symptom, as pain reduction is more important.	101 (56.4)	102 (57.0)	0.6
8. Palliative care requires constant emotional closeness.	32 (17.9)	46 (25.7)	7.8
9. Older people have learned to cope with grief on their own through various experiences of loss.	112 (62.6)	127 (70.9)	8.3
10. The philosophy of Palliative Care implies not carrying out interventions to prolong life.	62 (34.6)	91 (50.8)	16.2
11. The pain threshold is lowered by anxiety or fatigue.	59 (33.0)	84 (46.9)	13.9
12. Patients with life-threatening illnesses should always know the truth so that they can prepare for death.	33 (18.4)	59 (33.0)	14.6
13. Team members don’t need to have a particular belief or religion to provide spiritual care to end-of-life patients.	140 (78.2)	171 (95.5)	17.3
14. The patient receiving Palliative Care must accept death.	125 (69.8)	130 (72.6)	2.8
15. Communication skills can be learned.	177 (98.9)	179(100.0)	1.1
16. The death of a patient should not be communicated to other patients close to them and in a similar situation, to avoid disquiet.	37 (20.7)	56 (31.3)	10.6
17. The medical aspects of treatment always take priority in Palliative Care.	115 (64.2)	153 (85.5)	21.3
18. When a patient dies, visible ceremonies and ostentatious rites should be avoided to prevent disquiet in other patients.	57 (31.8)	77 (43.0)	11.2
19. The use of antidepressants to treat pain is not recommended.	142 (79.3)	174 (97.2)	17.9
20. Adjuvant analgesics are not necessary when the patient is receiving opioids.	138 (77.1)	165 (92.2)	15.1
21. The final phase includes the last three days of life.	39 (21.8)	68 (38.0)	16.2
22. The caregiver’s personal feelings (e.g. disgust) can come out while caring for patients.	106 (59.2)	122 (68.2)	9.0
23. Physiological needs (e.g. sexuality) are still important in the final phase of life.	115 (64.2)	135 (75.4)	11.2

*n (%) = Number (percentage);^†^Diff (%) = Percentage difference

**Table 4:** Evaluation of each pre-test and post-test self-efficacy item by nursing staff of the Bonn Palliative Care Knowledge Test (n = 179). Uberaba, MG, Brazil, 2024

Part 2 - Self-efficacy			
I believe I’m capable of...	Pre-test	Post-test	Diff (%)^†^
	n (%)*	n (%)*	
24. Collect information that objectively describes the intensity of the patient’s pain.	164 (91.6)	175 (99.5)	7.9
25. Orient the patient on how to relieve nausea.	153 (85.5)	176 (98.4)	12.9
26. Informing patients and their families about palliative care in the institution.	122 (68.2)	153 (85.4)	17.2
27. Convince the family doctor of the need for palliative care.	75 (41.9)	94 (52.6)	10.7
28. Identify and discuss concrete problems in the patient’s social context.	126 (70.4)	140 (78.2)	7.8
29. Arrange contact with a palliative care unit.	105 (58.7)	122 (68.2)	9.5
30. Talk to an anxious patient and their family to make them feel safe.	165 (92.1)	166 (92.7)	0.6
31. Identify the multiple needs of a patient at the end of life and react appropriately.	162 (88.9)	162 (88.9)	0.0
32. Offer appropriate relaxation alternatives to a patient in pain.	157 (87.7)	158 (88.2)	0.5
33. Talking to a patient who expresses the wish to anticipate death.	123 (68.7)	134 (74.8)	6.1
34. Carrying out appropriate oral hygiene for end-of-life patients.	178 (99.4)	179 (100.0)	0.6
35. Advising the patient on the possible side effects of the prescribed medication.	169 (94.4)	175 (97.8)	3.4
36. Identify specific psychological problems of two patients.	134 (74.9)	157 (87.7)	12.8
37. Integrating cultural aspects related to death and dying when caring for patients at the end of life.	119 (66.5)	143 (79.9)	13.4
38. Empathize with the needs of patients and their families in different life situations and react appropriately to them.	174 (97.3)	176 (98.3)	1.0

*n (%) = Number (percentage); ^†^Diff (%) = Percentage difference

## Discussion

Analysis of the collected data indicated a predominance of postgraduate courses in intensive care and in urgent and emergency care. This focus on curing and treating disease may lead nursing staff to overlook the importance of early integration and the benefits of a palliative approach combined with disease-modifying therapy^1^.

Competencies in PC are essential for professionals to provide high-quality care. They need to understand that, just as crucial as performing technical procedures, is developing skills such as symptom assessment and control, adequate communication and an understanding of the proportionality of care, which varies according to the patient’s clinical condition and capacity for recovery^14^.

These skills are increasingly important in a changing epidemiological scenario, where demand for PC is growing^2^. Studies on educational interventions contribute significantly to strengthening the scientific evidence of their benefits and to highlighting the relevance of these proposals for improving knowledge. Furthermore, they can promote changes in attitudes and beliefs and reduce barriers to health services, resulting in a better patient experience^15^.

In this study, an educational intervention in PC proved effective in increasing nursing professionals’ knowledge. A similar result was observed in a study involving 102 nurses, which indicated that in-service education improves professionals’ competence in providing qualified PC services. However, it was found that basic knowledge of PC remains limited. In this sense, learning interventions, such as continuing education in nursing, can promote significant advances in understanding PC and help overcome gaps in professional knowledge and skills^16^.

It is believed that changes in professional behavior occur when internal and external needs are transformed into motivation and purpose, mediated by self-awareness and understanding. Health service education promotes critical reflection on work processes, encouraging the problematization of service realities and the construction of solutions, integrating training, management, and care to improve practices, strengthen team performance, and enhance the quality of care for the community^17^. Thus, it is noted that integrating education into health services is essential to promote effective change in professional practice.

In this study, the mean value for the knowledge construct in PC on the pretest was low; however, regarding self-efficacy, the professionals reported feeling capable of providing this type of care. Similar studies, both national and international, corroborate these results^18^ ^-^ ^19^. However, it is not surprising that individuals consider themselves competent even with significant knowledge gaps, as there is a substantial difference between having knowledge and being able to use it effectively in various circumstances. Self-efficacy influences performance: thus, a person may consider themselves competent based on the feedback they receive, which may not be directly related to their level of knowledge^20^.

Regarding the difference in self-efficacy scores between the pre-test and post-test, a low gain was observed. This result may be related to the small-scale range (one to four) and the lack of a methodology that addresses the practical aspects of the subject. Thus, to have a more positive impact on self-efficacy in PC, it is essential to use different active learning methods, such as clinical case simulations. This strategy allows participants to learn how to correctly assess patients, identify their needs and develop a care plan. As a result, professionals tend to feel more self-confident in performing the tasks and demands assigned to them^21^.

When analyzing the instrument items separately within the knowledge construct, it is important to note that the medication-use questions (items 2, 4 and 19) showed the most significant increase in correct responses in the post-test. As these questions require more technical knowledge, it is considered that these items posed a challenge during the pre-test for the nursing team that responded to the questionnaire.

In this sense, it is emphasized that having technical knowledge, skills and a sense of being able to provide care in a palliative approach, especially in pain control, is essential for nursing professionals. Pain management in PC involves a comprehensive assessment of physical, psychological, social and spiritual determinants and combines pharmacological and non-pharmacological approaches. The nursing team must perform a thorough evaluation, incorporating patient behaviors and subjective and objective data, to adequately communicate pain levels to the interdisciplinary team and develop an effective management plan. However, barriers such as lack of knowledge of pain assessment and management, poor communication between professionals and the absence of standardized guidelines can compromise the adequate recognition of the patient’s perception, making technical preparation and reflective practice by the healthcare team essential^22^.

In this study, item 1 also yielded more correct responses in the post-test, indicating integration of PC with curative treatment. It is believed that during the pre-test, confusion arose due to professionals’ lack of understanding of the updated concept of PC. This result is of great importance for clinical practice, since early integration of this approach promotes advanced care planning, addressing issues such as pain control, management of physical symptoms, and psychosocial and spiritual support, which is reflected in the patient’s therapeutic success^23^.

A study conducted in the Netherlands found that when PC integration occurs late, during the end-of-life phase, patients are admitted with severe and difficult-to-control symptoms due to a lack of timely recognition of their needs^24^. This delay in eligibility is due to several barriers, such as stigma around referrals with an uncertain prognosis, difficulty in determining the criteria for identifying patients most likely to benefit from early follow-up, and a lack of knowledge among professionals on the subject^24^ ^-^ ^25^. These findings highlight the importance of knowledge and the need for discussion about PC, both in academia and among professionals already working in health institutions.

For self-efficacy items, there is no parameter for how true the statement is, since it concerns what the professional believes about themselves, which is a more subjective perception. However, it is expected that the responses affirm the veracity of the items, indicating that they feel capable of facing certain situations^26^. In the present study, the item with the highest gain in self-efficacy was item 26: “Inform the patient and family members about palliative care at the institution.” This finding reinforces the importance of disseminating PC in the current hospital environment, where the subject is so rarely addressed that it impedes the nursing team’s awareness of the interdisciplinary PC group’s presence in the institution. Thus, the importance of this research in addressing a gap in the study is evident.

The role of nurses in caring for patients with PC needs is associated with several essential nursing practices in multiple areas, such as direct patient and family care, leadership and environment management, as well as ensuring patient safety. However, there is a greater need for nurses involved in research activities specifically focused on the topic of PC, which are essential for evidence-based practice. Thus, there is an opportunity to fill gaps through scientific publications, since by addressing recommendations related to research, education and public policy, stakeholders can work to strengthen the role of nurses in PC and improve the outcomes of care provided to patients and their families^27^.

A limitation of the study is the absence of practical teaching associated with theoretical content during the educational intervention, which would have helped to achieve more significant benefits, not only in terms of knowledge acquisition, but mainly in terms of self-efficacy, assisting the professionals to feel more secure and confident in applying the knowledge acquired in their professional practice. In addition, the time allocated to the educational intervention was considered insufficient for an in-depth exploration of all aspects of the topic.

These results indicate an increase in assertive responses between the pre-test and post-test, reinforcing the study’s relevance and the need for educational interventions within the health service for the nursing team. This shows that educational intervention is an essential tool in the teaching and learning process for this topic. It should be noted that implementing these care measures can mitigate patients’ and their families’ suffering, providing greater comfort and quality of care, thereby reducing therapeutic obstinacy.

The study contributes significantly to advancing knowledge in the field of PC by demonstrating the effectiveness of an educational intervention. It may contribute to more planned, targeted nursing care, including guidance, dialogue and health behaviors, for this specific population. In the scientific and academic sphere, the research strengthens the evidence base for nursing educational interventions, encouraging new studies aimed at humanizing care and expanding comprehensive, effective access to PC.

Thus, nursing professionals must be willing to apply the available evidence from the literature in practice to achieve the best health outcomes. Professional practice in PC must be supported by a solid structure of knowledge, ethics, principles, application of the scientific method and development of a care plan in the process of critical and clinical thinking, with a focus on decision-making and problem-solving, interfering in the improvement of the quality of life, comfort and dignity of patients and their respective families and caregivers.

## Conclusion

The educational intervention was effective in increasing knowledge and self-efficacy in PC among nursing professionals, with a significant improvement observed, especially in knowledge. In terms of gaining knowledge, the highlight was medication issues for pain management. Regarding self-efficacy, the most essential gain was observed in the item related to professionals referring patients and their families to PC services at the institution itself.

These results can support the development and implementation of educational interventions, specifically in PC and in health services, to minimize the gaps identified as relevant in this approach.

## Data Availability

All data generated or analysed during this study are included in this published article.
