# Supplementary material for: Educational intervention for nursing professionals on palliative care: quasi-experimental study
Source: Rev Lat Am Enfermagem. 2026 Jun 15;34:e4822. doi: 10.1590/1518-8345.8026.4822 (PMC13277347; doi:10.1590/1518-8345.8026.4822)
Supplement: Translation

# Translation: Intervenção educativa aos profissionais de enfermagem sobre cuidados paliativos: estudo quase experimental*

**DOI:** 10.1590/1518-8345.8026.4823

**Authors:** Joyce Assunção Barros, Taciana Fernandes Araújo Ferreira, Fabiana Bolela, Raquel Pan, Silmara Elaine Malaguti Toffano, Adriana Cristina Nicolussi

**Affiliations:** 1 Universidade Federal do Triângulo Mineiro, Uberaba, MG, Brasil.; 2 Bolsista da Fundação de Amparo à Pesquisa do Estado de Minas Gerais (FAPEMIG), Brasil.; 3 Universidade de São Paulo, Escola de Enfermagem de Ribeirão Preto, Centro Colaborador da OPAS/OMS para o Desenvolvimento da Pesquisa em Enfermagem, Ribeirão Preto, SP, Brasil.

**Keywords:** Cuidados Paliativos, Equipe de Enfermagem, Educação Continuada em Enfermagem, Conhecimento, Autoeficácia, Atenção Terciária à Saúde

## Abstract

**Objetivo:**

avaliar o conhecimento e a autoeficácia sobre cuidados paliativos de profissionais de enfermagem antes e depois de uma intervenção educativa.

**Método:**

estudo quase experimental, realizado em um hospital de ensino, com profissionais de enfermagem que atendem ao público adulto e idoso. A coleta de dados foi realizada em dois momentos: no primeiro, foram aplicados o questionário sociodemográfico e o instrumento *Bonn Palliative Care Knowledge Test*, que avalia o conhecimento e a autoeficácia em cuidados paliativos; logo após, ocorreu a intervenção educativa sobre a temática. No segundo momento, após quatro semanas, foi realizada a reaplicação do instrumento que avalia o conhecimento e a autoeficácia. Os dados foram avaliados no programa *Statistical Package for the Social Sciences* versão 21.0.

**Resultados:**

participaram 179 profissionais, com média de idade de 40,17 anos e predominância do sexo feminino. O escore de conhecimento obteve médias mais altas após a intervenção (pré-teste=11,77; pós-teste=15,03) enquanto o da autoeficácia foi em menor escala (pré-teste=3,21; pós-teste=3,38), contudo ambas foram estatisticamente significativas (p<0,01).

**Conclusão:**

a intervenção educativa foi eficaz para o ganho de conhecimento e autoeficácia em cuidados paliativos nos profissionais de enfermagem. Os referidos resultados podem subsidiar a elaboração e disseminação das práticas de cuidados paliativos nos serviços de saúde.

## Destaques

**(1)** A intervenção educativa teve um impacto positivo no conhecimento dos profissionais. **(2)** O número de respostas corretas após a intervenção educativa aumentou. **(3)** O processo de ensino-aprendizagem em cuidados paliativos deve ser incentivado. **(4)** Os resultados podem subsidiar a elaboração de outras intervenções educativas. **(5)** A implementação desses cuidados pode proporcionar maior qualidade na assistência.

## Introdução

Os Cuidados Paliativos (CP) são destinados às pessoas com doenças ou condições que ameaçam a vida e ocasionam limitações e perda progressiva da funcionalidade. Têm como benefícios e objetivo principal prevenir e tratar o sofrimento que o paciente, família e o cuidador possam vivenciar, seja ele de natureza física, psicológica, social e/ou espiritual^1^. 

Apesar de muitos avanços no Brasil, os CP ainda são pouco difundidos, tanto na assistência em serviços especializados quanto na área da educação, visto que existem falhas no conhecimento dos profissionais de saúde, falta de treinamento com as equipes já atuantes e ausência do conteúdo na matriz curricular dos ensinos de graduação^2^.

Análise sobre a compreensão dos profissionais de saúde acerca de CP evidenciou que eles possuem pouco conhecimento ou têm uma visão distorcida sobre o tema. Tais dados são preocupantes, pois a falta de capacitação gera insegurança em relação a como agir em situações específicas, privando pacientes e familiares de receberem os benefícios que os CP podem proporcionar^3^. 

O Ministério da Saúde (MS) brasileiro vem contribuindo para a implementação dessa abordagem no país. Em sete de dezembro de 2023, foi publicada a Resolução n.º 729, que aprovou a Política Nacional de Cuidados Paliativos (PNCP) no Sistema Único de Saúde (SUS), que prevê a garantia de acesso a essa assistência em qualquer ponto da Rede de Atenção à Saúde (RAS). Diante desse cenário, entende-se que a demanda relacionada aos cuidados desses pacientes não será suprida apenas por equipes especializadas em CP, pois apresentam número insuficiente no Brasil. Dessa forma, os profissionais não especialistas, já atuantes nas RAS, seriam os responsáveis por cuidar da maior parte das demandas relacionadas aos CP^4^.

Porém, para que isso aconteça, estes profissionais precisam desenvolver conhecimento e competências sobre a temática, para assim aplicar esta prática corretamente, seguindo os princípios e oferecendo assistência com qualidade, sem deixar a população desassistida. Profissionais de enfermagem são responsáveis por cuidar da maior parte das demandas; com isso, tornam-se necessárias ações de educação como parte do planejamento do sistema de saúde, para que se favoreça não só o acesso, mas a resolutividade das necessidades de saúde dos usuários que precisam desses cuidados^5^.

A educação em serviço é um processo contínuo e dinâmico para a construção do saber profissional. Atividades educativas proporcionam pensamento crítico e transformação da realidade em que o profissional está inserido. Aliado a isso, as práticas de enfermagem estão sempre associadas às ações educativas, promovendo mudanças em suas atividades, garantindo a qualidade da assistência e o contínuo aprimoramento profissional^6^.

Assim, tendo em vista o baixo desenvolvimento dos CP no Brasil, possivelmente associado às lacunas no conhecimento dos profissionais e considerando que se trata de uma prática essencial nos sistemas de saúde, principalmente nesse contexto de transição sociodemográfica, com uma crescente demanda de pacientes que podem se beneficiar dessa abordagem, torna-se relevante o desenvolvimento de ações voltadas à construção de conhecimento e à sensibilização sobre o tema, com vistas a qualificar a assistência prestada à população^5^.

Nesse contexto, identificar o que o profissional possui de conhecimento deve ser considerado na prática de ensino, pois facilita o processo de aprendizagem e sua transmutação em ações práticas para mudança de realidade^7^. Dado o cenário exposto, este estudo objetivou avaliar o conhecimento e a autoeficácia sobre CP de profissionais de enfermagem antes e depois de uma intervenção educativa.

## Método

### Delineamento do estudo

Este é um estudo quase experimental, do tipo antes e depois, no qual os mesmos sujeitos foram avaliados no pré-teste e no pós-teste de uma intervenção educativa, que seguiu a lista de verificação *Template for Intervention Description and Replication* (TIDieR).

### Local

A pesquisa foi realizada em um hospital público de ensino, na cidade de Uberaba, no estado de Minas Gerais (MG), Brasil. O hospital possui 302 leitos ativos e atende à demanda do SUS de uma macrorregião de Minas Gerais. A instituição é referência para a média e alta complexidade^8^.

### Período

A coleta de dados foi realizada no período de maio a setembro de 2024.

### Amostra e critérios de inclusão

A população do estudo foi composta por profissionais de enfermagem (enfermeiros, técnicos em enfermagem e auxiliares de enfermagem) com idade igual ou superior a 18 anos, que atuavam na assistência direta ao paciente adulto/idoso, dos seguintes setores em regime de internação: ortopedia, clínica médica, clínica cirúrgica, unidade de doenças infecciosas e parasitárias, neurologia, ginecologia e obstetrícia, onco-hematologia, unidade de terapia intensiva I, II e coronária e pronto-socorro adulto. Foram excluídos os profissionais que estavam de férias ou afastados do trabalho no período da realização do estudo.

O cálculo do tamanho amostral foi realizado após análise do estudo-piloto com 18 profissionais. O nível de significância considerado foi um erro do tipo I (α=0,01), um erro do tipo II (β=0,1) e um poder estatístico de 90%. Considerando o conhecimento em CP como o desfecho primário, a média de aumento dos escores do pré-teste para o pós-teste foi de 5,72 pontos (dp=3,10), o que equivale a um ganho superior a 50%. Com base nesses valores, o tamanho amostral foi realizado por meio do programa *Power Analysis and Sample Size* (PASS), resultando em um total de 30 participantes. Salienta-se que os participantes do estudo-piloto não fizeram parte do estudo principal.

Tendo em vista que a magnitude do efeito da intervenção no estudo-piloto foi maior do que o esperado, decidiu-se, por precaução, que o estudo principal deveria incluir um número de participantes superior ao preconizado no estudo-piloto. Como a intervenção foi realizada em parceria e cadastrada no Serviço de Educação em Enfermagem (SEE) do referido hospital, optou-se por convidar todos os profissionais de enfermagem interessados em participar do estudo. Portanto, utilizou-se uma amostragem não probabilística, do tipo por conveniência.

Dessa forma, participaram do estudo no pré-teste 214 profissionais de enfermagem; entretanto, na etapa da aplicação do pós-teste, houve perda de 35 participantes, sendo a amostra final de 179 profissionais.

### Instrumentos de coleta de dados

Utilizou-se para caracterizar a amostra um questionário sociodemográfico e profissional elaborado pelos próprios autores, contendo as variáveis: idade, sexo, grau de escolaridade, categoria profissional de atuação, titulação, especialização (pós-graduação), setor de lotação, tempo total de experiência profissional e o turno de trabalho.

Para a avaliação do conhecimento e autoeficácia em CP, foi utilizado o instrumento *Bonn Palliative Care Knowledge Test* (BPW), desenvolvido na Alemanha em 2011^9^, traduzido e adaptado culturalmente para o contexto brasileiro em 2018^10^. Ele contém 38 questões, sendo 23 destinadas à avaliação do conhecimento, com tópicos sobre o controle da dor e outros sintomas, o conhecimento geral sobre o tema e as atitudes ante a morte e o morrer. De acordo com as instruções do instrumento, os itens de conhecimento numerados como 1 a 4, 6 a 10, 12, 14 e 16 a 20 devem ser considerados incorretos, enquanto os itens 5,11,13,15,21 a 23 são considerados corretos.

As 15 questões restantes avaliam a autoeficácia na prestação de CP e fornecem informações sobre confiança do participante para implementar o que foi aprendido na prática clínica. As questões são organizadas no formato de uma escala tipo Likert, cujas respostas incluem “correto”, “mais correto que incorreto”, “mais incorreto que correto” ou “incorreto”^9^ ^-^ ^10^.

Para a escala do conhecimento, o escore geral representa a soma dos itens considerados corretos e pode variar de zero (se o respondente errou todos os itens) a 23 (se acertou todos). Quanto maior o escore, maior o conhecimento do profissional. Na escala de autoeficácia, o escore geral representa a média das respostas aos itens da escala e pode variar de zero (sente-se incapaz) a quatro (sente-se totalmente capaz), ou seja, quanto mais próximo de quatro, mais o profissional sente-se capaz para realizar determinada ação descrita no instrumento^9^ ^-^ ^10^.

### Etapa operacional

O conteúdo programático da aula da intervenção educativa foi desenvolvido com base no manual de CP do Ministério da Saúde em parceria com o Hospital Sírio-Libanês (2023)^1^ e na literatura vigente, com foco nas questões importantes para o conhecimento em CP.

Sendo assim, os tópicos de abordagem selecionados e elaborados foram: a) conceito atualizado e os princípios de CP; b) a importância do tema para os profissionais de saúde, com apresentação de dados epidemiológicos; c) a relação dos CP com tratamento modificador da doença, destacando que os objetivos da terapêutica paliativa estarão de acordo com a evolução funcional e clínica de cada paciente; d) a importância da assistência multiprofissional; e) identificação e controle dos sintomas físicos prevalentes nesta demanda (dor, náuseas e vômitos, constipação e dispneia); f) os principais sintomas psíquicos (ansiedade, depressão e *delirium*); g) os aspectos sociais mais comuns, como perda da identidade social, perda do papel social e familiar e isolamento social; h) espiritualidade; i) cuidados de fim de vida, destacando que os tratamentos propostos devem ser avaliados em relação à sua proporcionalidade ante o processo de finitude; j) comunicação efetiva entre equipe, paciente e família; k) os serviços que estão disponíveis no hospital para atender à demanda de CP e como realizar a solicitação desse serviço.

Após a elaboração da aula, o conteúdo foi enviado para validação, que ocorreu de forma independente, por meio de uma carta-convite, via eletrônica (e-mail), para 13 doutores previamente selecionados por meio da análise do Currículo Lattes, que possuíam conhecimento sobre o constructo em questão. Dos 13 doutores, sete não responderam a nenhum dos contatos eletrônicos e três recusaram participar. Assim, três doutores fizeram parte da validação do conteúdo.

Para aqueles que concordaram em participar da validação, foi enviado um novo *e-mail* contendo o Termo de Consentimento Livre e Esclarecido (TCLE) e o Instrumento de Validação de Conteúdo Educativo em Saúde (IVCES). O prazo solicitado para devolução do instrumento pelo especialista aos pesquisadores foi de 10 dias, podendo esse período ser flexibilizado, caso necessário.

O IVCES possui 18 itens, sendo dividido em três grupos: objetivos (1-5), estrutura e apresentação (6-15) e relevância (16-18), com opções de respostas utilizando a escala Likert, sendo 0 = discordo, 1 = concordo parcialmente e 2 = concordo totalmente. Além da pontuação do instrumento, o especialista podia também registrar em texto corrido, caso necessário, suas considerações^11^. 

Após o preenchimento e a devolução do instrumento por todos os especialistas, procedeu-se à avaliação da concordância entre os juízes quanto à validação do conteúdo. Para isso, foi calculado o Índice de Validade de Conteúdo (IVC), que corresponde à divisão entre a soma do número de respostas em concordância (itens marcados como 2) pelo total de respostas, considerando como concordância aceitável o valor mínimo recomendável de 80%.

O especialista 1 atribuiu a pontuação máxima (100%), assinalando a opção “concordo totalmente” para todos os 18 itens do instrumento. Já os especialistas 2 e 3 obtiveram um índice de 80%, com 15 itens avaliados como “concordo totalmente” e 3 como “concordo parcialmente”.

Salienta-se que esse processo de validação é importante, pois avalia a clareza e o entendimento do público-alvo, sua representatividade ao abordar adequadamente o universo a que se propõe e, ainda, busca evitar a inclusão de elementos desnecessários^11^.

Após a validação do conteúdo, a intervenção educativa foi cadastrada no SEE do referido hospital. Foram realizadas reuniões com os pesquisadores, a enfermeira do SEE e os Responsáveis Técnicos (RT) de cada setor participante da pesquisa, com o objetivo de definir e agendar as datas e os horários das aulas. Os RT ficaram responsáveis pela divulgação e direcionamento da equipe de enfermagem para a participação na intervenção.

Em seguida, a intervenção educativa foi testada em um estudo-piloto com 18 profissionais e, então, foram realizadas adequações apenas em relação à estrutura da apresentação, sem alteração no conteúdo, a fim de otimizar o tempo da intervenção no horário programado. Após a realização do piloto, foi iniciada a fase da intervenção educativa do estudo principal.

### Coleta de dados

A intervenção educativa foi realizada por meio de apresentação expositiva com o apoio do recurso visual Microsoft Power Point^®^ e utilizou também a metodologia participativa e dialógica, com o objetivo de permitir a troca de experiências, esclarecimento de dúvidas e discussão das questões levantadas pelos participantes sobre o tema.

A coleta de dados foi realizada em dois momentos (pré-teste e pós-teste). O primeiro, de forma coletiva e em pequenos grupos com as equipes de enfermagem, em sala de aula reservada no próprio hospital, com agendamento prévio das datas e horários dentro do expediente de trabalho dos profissionais, nos três turnos (matutino, vespertino e noturno). Foram necessárias várias disponibilidades de horários e dias de aula devido à impossibilidade de saída dos profissionais ao mesmo tempo de cada setor, sendo que, no total, foram ofertadas 51 aulas.

Foram apresentados em cada encontro os objetivos da pesquisa, bem como foi lido o TCLE. Após aceitação e assinatura, foram aplicados o questionário sociodemográfico e profissional e o instrumento BPW. Estabeleceu-se um tempo aproximado de vinte minutos para o preenchimento dos instrumentos, os quais foram preenchidos individualmente. Esse tempo está de acordo com o preconizado pelos autores do estudo original do instrumento e foi determinado também com base no estudo-piloto. Em seguida, ocorreu a educação em serviço de saúde em CP, conduzida pelos próprios pesquisadores. A aula teve duração de aproximadamente quarenta minutos, totalizando cerca de uma hora para toda a atividade.

No segundo momento da coleta de dados, quatro semanas após a intervenção educativa, foi realizada a reaplicação apenas do instrumento BPW. Essa etapa ocorreu de forma individual, por meio de busca ativa dos participantes nos próprios setores em que atuavam. O preenchimento do instrumento teve duração aproximada de dez minutos.

### Tratamento e análise dos dados

Os dados coletados foram inseridos em uma planilha eletrônica Excel^®^ do programa Windows^®^ por meio da técnica de dupla digitação, para posterior validação. A identificação de cada participante do estudo ocorreu por meio de códigos. Em seguida, o banco de dados validado foi importado no programa *Statistical Package for the Social Sciences* (SPSS) versão 21.0 para processamento e análise^12^.

Os dados foram analisados por meio de estatística descritiva com medidas de tendência central (média) e de dispersão (desvio-padrão) para as variáveis quantitativas e frequências absolutas e relativas para as categóricas. Foi avaliada a diferença das médias entre o pré-teste e o pós-teste tanto do conhecimento quanto da autoeficácia em CP. Todos os requisitos para a utilização de testes paramétricos foram devidamente verificados, incluindo a normalidade dos dados e a análise de valores atípicos. Sendo assim, foi aplicado o teste T pareado, nível de significância I α=0,01, considerando intervalo de confiança de 99%.

Para verificar a magnitude do efeito da intervenção, utilizou-se um aplicativo disponibilizado no site Psychometrica.de (https://www.psychometrica.de/effect_size.html)^13^. A interpretação dos tamanhos de efeito seguiu a seguinte classificação de Cohen: d de Cohen (0,0 a 0,1) = sem efeito; d de Cohen (0,2 a 0,4) = efeito pequeno; d de Cohen (0,5 a 0,7) = efeito moderado e d de Cohen (0,8 a ≥1,0) = efeito grande.

### Aspectos éticos

A pesquisa foi registrada na Rede Pesquisa, ferramenta que compõe a Rede da Empresa Brasileira de Serviços Hospitalares (EBSERH), sob o código n.^o^ 3738, obteve o assentimento da Gerência de Ensino e Pesquisa do referido hospital e foi aprovada pelo Comitê de Ética em Pesquisa da instituição proponente, parecer n.^o^ 6.510.776, respeitando os princípios éticos.

## Resultados

Dos 179 participantes avaliados neste estudo, a maioria (82,1%) era do sexo feminino, com média de idade de 40,17 anos (dp = 8,5), sendo a mínima 23 e a máxima 64 anos. 

Quanto aos dados profissionais, a maioria (69,3%) foi composta por técnicos em enfermagem, predominando os que atuavam no setor da clínica médica (26,3%) e turno noturno (36,3%), com tempo de experiência profissional de mais de 10 anos (64,2%). No que se refere à pós-graduação, 44,1% relataram ter feito alguma especialização, prevalecendo cursos nas áreas de cuidados intensivos (30,37%) e urgência e emergência (26,58%). Apenas um participante relatou possuir pós-graduação na área de CP.

Em relação ao escore de conhecimento e autoeficácia em CP, houve um aumento no número de respostas corretas após a intervenção educativa, conforme mostra a Tabela 1.

**Tabela 1:** Distribuição dos escores da amostra aferidos pelo *Bonn Palliative Care Knowledge Test* (n = 179). Uberaba, MG, Brasil, 2024

Variáveis	Média	Dp*	Mínimo	Máximo
Escore de conhecimento pré-teste	11,77	2,56	4	18
Escore de conhecimento pós-teste	15,03	3,64	8	23
Escore de autoeficácia pré-teste	3,21	0,48	1,73	4
Escore de autoeficácia pós-teste	3,38	0,43	2	4

^*^ Dp = Desvio-padrão

Quanto aos constructos do conhecimento, as médias aumentaram consideravelmente após a educação em serviço, com um ganho de três pontos na média de desempenho. Já os resultados referentes à autoeficácia obtiveram aumento, porém, em uma menor escala, com um ganho apenas de 0,1. Ambas foram consideradas estatisticamente significativas (p<0,01). Em relação ao efeito da magnitude da intervenção, o teste de conhecimento foi considerado grande (0,8 a 1,0) e o de autoeficácia obteve efeito moderado (0,5 a 0,7), conforme demonstrado na Tabela 2. Vale ressaltar que, para as avaliações do instrumento, quanto mais próximo de 23, maior o conhecimento e quanto mais próximo de quatro, maior a autoeficácia percebida.

**Tabela 2:** Comparação da diferença das médias entre o pré-teste e o pós-teste da equipe de enfermagem do *Bonn Palliative Care Knowledge Test* (n = 179). Uberaba, MG, Brasil, 2024

	Teste de amostras pareadas			
	Média	Dp*	*p* ^†^	d^‡^
Escore de conhecimento pré e pós-teste	-3,26	4,10	0,000	0,83
Escore de autoeficácia pré e pós-teste	-0,17	0,49	0,000	0,52

*Dp = Desvio-padrão; ^†^Valor de p = Significativo <0,001; ^‡^d = d de Cohen

A Tabela 3 apresenta os resultados do pré-teste e pós-teste de cada item da avaliação de conhecimento do BPW e a Tabela 4, os da avaliação da autoeficácia. A questão que apresentou um ganho maior de acertos após a intervenção foi o item dois (diferença percentual de 35,2%), que se refere ao uso de medicamentos anti-inflamatórios com o uso de opioides. Já nos dados referentes à autoeficácia, houve uma menor variância de amplitude entre o pré-teste e o pós-teste, pois a escala varia apenas de um a quatro e ressalta-se que, no pré-teste, os resultados foram próximos do valor limite. Dentre os itens que tiveram maior ganho após educação em serviço, destacou-se o item 26 (diferença percentual de 17,2%), que aborda a informação sobre CP na própria instituição.

**Tabela 3:** Avaliação de cada item do conhecimento pré-teste e pós-teste da equipe de enfermagem do instrumento *Bonn Palliative Care Knowledge Test* (n = 179). Uberaba, MG, Brasil, 2024

Questões	Respostas corretas		
	Pré-teste	Pós-teste	
Parte 1 - Conhecimento	n (%)*	n (%)*	Dif (%)^†^
1. Cuidados paliativos nunca devem ser combinados com tratamentos curativos.	120 (67,0)	161 (89,9)	22,9
2. Medicamentos anti-inflamatórios não esteroides não devem ser utilizados durante o uso regular de opioides.	91 (50,8)	154 (86,0)	35,2
3. Administração de fluidos por via subcutânea (hipodermóclise) é necessária para aliviar a boca seca (xerostomia) em pacientes em fase final de vida.	97 (54,2)	132 (73,7)	19,7
4. O uso de adesivos transdérmicos para controle da dor instável é adequado em pacientes em fase final de vida.	55 (30,7)	102 (57,0)	26,3
5. Terapias adjuvantes (por ex., fisioterapia) são importantes para o controle da dor.	142 (79,3)	156 (87,2)	7,9
6. Para os familiares, é sempre importante permanecer ao lado do paciente que está morrendo.	13 (7,3)	48 (26,8)	18,9
7. A constipação deve ser aceita como um sintoma secundário, pois a redução da dor é mais importante.	101 (56,4)	102 (57,0)	0,6
8. Os cuidados paliativos requerem proximidade emocional constante.	32 (17,9)	46 (25,7)	7,8
9. Pessoas com idade avançada aprenderam com várias experiências de perda a lidar com o luto, por si só.	112 (62,6)	127 (70,9)	8,3
10. A filosofia dos Cuidados Paliativos implica não realizar intervenções para prolongar a vida.	62 (34,6)	91 (50,8)	16,2
11. O limiar da dor é reduzido pela ansiedade ou fadiga.	59 (33,0)	84 (46,9)	13,9
12. Pacientes com doenças que ameaçam a vida devem sempre saber da verdade para que possam se preparar para a morte.	33 (18,4)	59 (33,0)	14,6
13. Os membros da equipe não precisam ter uma determinada crença ou religião para proporcionar cuidado espiritual aos pacientes em fase final de vida.	140 (78,2)	171 (95,5)	17,3
14. O paciente que recebe Cuidados Paliativos deve aceitar a morte.	125 (69,8)	130 (72,6)	2,8
15. Habilidades de comunicação podem ser aprendidas.	177 (98,9)	179 (100,0)	1,1
16. A morte de um paciente não deve ser comunicada para outros pacientes próximos dele e em situação semelhante para evitar inquietação.	37 (20,7)	56 (31,3)	10,6
17. Os aspectos médicos do tratamento sempre têm prioridade nos Cuidados Paliativos.	115 (64,2)	153 (85,5)	21,3
18. Quando um paciente morre, devem ser evitadas cerimônias visíveis e ritos ostensivos para prevenir a inquietação nos outros pacientes.	57 (31,8)	77 (43,0)	11,2
19. O uso de antidepressivos para o tratamento da dor não é recomendável.	142 (79,3)	174 (97,2)	17,9
20. Analgésicos adjuvantes não são necessários quando o paciente recebe opioides.	138 (77,1)	165 (92,2)	15,1
21. A fase final compreende os últimos três dias de vida.	39 (21,8)	68 (38,0)	16,2
22. Os sentimentos pessoais do cuidador (por exemplo, aversão) podem transparecer durante o cuidado de pacientes.	106 (59,2)	122 (68,2)	9,0
23. Necessidades fisiológicas (por exemplo, sexualidade) ainda são importantes na fase final de vida.	115 (64,2)	135 (75,4)	11,2

*n (%) = Número (porcentagem); ^†^Dif (%) = Diferença percentual

**Tabela 4:** Avaliação de cada item da autoeficácia pré-teste e pós-teste da equipe de enfermagem do instrumento *Bonn Palliative Care Knowledge Test* (n = 179). Uberaba, MG, Brasil, 2024

Parte 2 - Autoeficácia			
Acredito que sou capaz de...	Pré-teste	Pós-teste	Dif (%)^†^
	n (%)*	n (%)*	
24. Coletar informações que descrevam a intensidade da dor do paciente de maneira objetiva.	164 (91,6)	175 (99,5)	7,9
25. Orientar o paciente sobre como aliviar a náusea.	153 (85,5)	176 (98,4)	12,9
26. Informar o paciente e aos familiares sobre os cuidados paliativos na instituição.	122 (68,2)	153 (85,4)	17,2
27. Convencer o médico de família sobre as necessidades dos cuidados paliativos.	75 (41,9)	94 (52,6)	10,7
28. Identificar e discutir problemas concretos no contexto social do paciente.	126 (70,4)	140 (78,2)	7,8
29. Providenciar o contato com uma unidade de cuidados paliativos.	105 (58,7)	122 (68,2)	9,5
30. Conversar com um paciente ansioso e seus familiares para que se sintam seguros.	165 (92,1)	166 (92,7)	0,6
31. Identificar as múltiplas necessidades de um paciente em final de vida e reagir de forma apropriada.	162 (88,9)	162 (88,9)	0,0
32. Oferecer alternativas apropriadas de relaxamento a um paciente com dor.	157 (87,7)	158 (88,2)	0,5
33. Conversar com um paciente que expressa o desejo de antecipar a morte.	123 (68,7)	134 (74,8)	6,1
34. Realizar higiene oral apropriada de pacientes em final de vida.	178 (99,4)	179 (100,0)	0,6
35. Orientar o paciente sobre possíveis efeitos colaterais dos medicamentos prescritos.	169 (94,4)	175 (97,8)	3,4
36. Identificar problemas psicológicos específicos dos pacientes.	134 (74,9)	157 (87,7)	12,8
37. Integrar aspectos culturais relacionados à morte e o morrer ao cuidar de pacientes em final de vida.	119 (66,5)	143 (79,9)	13,4
38. Ter empatia com as necessidades de pacientes e familiares, em diferentes situações de vida, e reagir apropriadamente a elas.	174 (97,3)	176 (98,3)	1,0

*n (%) = Número (porcentagem); ^†^Dif (%) = Diferença percentual

## Discussão

A análise dos dados coletados indicou a predominância do curso de pós-graduação em cuidados intensivos e urgência e emergência. Tal foco na cura e no tratamento da doença pode influenciar a equipe de enfermagem a não compreender a importância da integração precoce e os benefícios de uma abordagem paliativa aliada à terapia modificadora da doença^1^. 

As competências em CP são essenciais para que os profissionais exerçam uma prática de alta qualidade. É necessário que compreendam que, tão fundamental quanto realizar procedimentos técnicos, é desenvolver habilidades como avaliação e controle de sintomas, comunicação adequada e entendimento da proporcionalidade do cuidado, que modifica de acordo com a condição clínica e a capacidade de recuperação do paciente^14^.

Essas habilidades são cada vez mais importantes em um cenário de mudança epidemiológica, no qual a demanda por CP é crescente^2^. Estudos sobre intervenções educativas contribuem significativamente para fortalecer as evidências científicas sobre seus benefícios e evidenciar a relevância dessas propostas no aprimoramento do conhecimento. Ademais, podem favorecer mudanças de atitudes, a modificação de crenças e a redução de barreiras nos serviços de saúde, resultando em uma melhor experiência do paciente^15^. 

Neste estudo, a intervenção educativa em CP mostrou-se eficaz no ganho de conhecimento dos profissionais de enfermagem. Resultado semelhante foi observado em pesquisa realizada com 102 enfermeiros, demonstrando que a educação em serviço contribui para aprimorar a competência dos profissionais na prestação de serviços qualificados em CP. Constatou-se, entretanto, que o conhecimento básico sobre CP ainda é limitado. Nesse sentido, intervenções de aprendizagem, como a educação continuada em enfermagem, podem promover avanços significativos na compreensão dos CP e auxiliar na superação de lacunas relacionadas ao conhecimento e às habilidades profissionais^16^.

Acredita-se que a modificação do comportamento dos profissionais ocorre quando necessidades internas e externas transformam-se em motivação e propósito, mediadas pela autoconsciência e compreensão. A educação em serviço de saúde promove a reflexão crítica sobre os processos de trabalho, favorecendo a problematização da realidade dos serviços e a construção de soluções, integrando formação, gestão e atenção para aprimorar práticas, fortalecer a atuação da equipe e melhorar a qualidade do cuidado à comunidade^17^. Dessa forma, nota-se que a inserção do processo educacional nos serviços de saúde é essencial para promover mudanças efetivas na prática profissional.

Verificou-se que, neste estudo, o valor médio para o constructo do conhecimento em CP no pré-teste foi baixo; já na autoeficácia, os profissionais responderam que se sentiam capazes de prestar esse tipo de cuidado. Estudos semelhantes, nacional e internacional, corroboram tais resultados^18^ ^-^ ^19^. Entretanto, não é surpreendente que indivíduos considerem-se competentes mesmo com grandes lacunas em conhecimento, pois há grande diferença entre possuir conhecimentos e ser capaz de utilizá-los bem, sob variadas circunstâncias. A autoeficácia exerce influência sobre performance; assim, uma pessoa pode se considerar competente em razão do *feedback* recebido, o que pode não ter relação direta com seu nível de conhecimento^20^.

Com relação à diferença de escore em autoeficácia entre o pré-teste e o pós-teste, percebeu-se um baixo ganho. Esse resultado pode estar relacionado à variância de amplitude pequena da escala (um a quatro) e à falta de uma metodologia que envolva parte prática no assunto abordado. Dessa forma, para ter um impacto mais positivo em autoeficácia em CP, é importante a utilização de diferentes métodos de aprendizagem ativa, como, por exemplo, a simulação de um caso clínico. Essa estratégia permite que os participantes aprendam a avaliar corretamente o paciente, identifiquem suas necessidades e elaborem um plano de cuidados. Com isso, os profissionais tendem a sentir-se mais autoconfiantes na realização das tarefas e demandas atribuídas^21^.

Ao analisar separadamente os itens do instrumento no constructo do conhecimento, é importante destacar que as questões referentes ao uso de medicamentos foram os itens que obtiveram um ganho significativo de acertos no pós-teste (itens 2, 4 e 19). Por serem questões que envolvem um conhecimento mais técnico, considera-se que os referidos itens tenham constituído um desafio no pré-teste para a equipe de enfermagem que respondeu ao questionário.

Nesse sentido, enfatiza-se que ter conhecimento técnico, habilidades e sentir-se capaz de prestar os cuidados em uma abordagem paliativa, especialmente no controle de dor, é essencial para os profissionais de enfermagem. O tratamento da dor em CP envolve uma avaliação integral, considerando determinantes físicos, psicológicos, sociais e espirituais e combina abordagens farmacológicas e não farmacológicas. A equipe de enfermagem deve realizar uma avaliação completa, incorporando comportamentos do paciente e informações subjetivas e objetivas, a fim de comunicar adequadamente os níveis de dor à equipe interdisciplinar e elaborar um plano de manejo eficaz. No entanto, barreiras como a falta de conhecimento sobre avaliação e manejo da dor, comunicação deficiente entre os profissionais e ausência de diretrizes padronizadas podem comprometer o reconhecimento adequado da percepção do paciente, tornando essencial o preparo técnico e a prática reflexiva da equipe de saúde^22^.

Verificou-se que, neste estudo, o item 1 também apresentou um maior número de respostas corretas no pós-teste, o qual se refere à integração de CP com o tratamento curativo. Acredita-se que, no pré-teste, tenha ocorrido confusão devido à falta de entendimento dos profissionais sobre o conceito atualizado de CP. Esse resultado tem grande importância para a prática clínica, uma vez que a integração precoce dessa abordagem promove o planejamento antecipado dos cuidados, abordando questões como controle da dor, manejo dos sintomas físicos e apoio psicossocial e espiritual, o que reflete no sucesso terapêutico do paciente^23^.

Estudo realizado na Holanda identificou que, quando a integração de CP ocorre tardiamente, já durante a fase fim de vida, os pacientes são internados com sintomas severos e de difícil controle, devido à falta de reconhecimento das necessidades em tempo hábil^24^. Esse atraso na elegibilidade deve-se às diversas barreiras, como estigmas relacionados ao encaminhamento devido a um prognóstico incerto, dificuldade em conhecer os critérios de identificação dos pacientes mais propensos a se beneficiarem do acompanhamento precoce e falta de conhecimento dos profissionais sobre o tema^24^ ^-^ ^25^. Esses achados ressaltam a importância do conhecimento e da necessidade de discussão sobre CP, tanto no meio acadêmico quanto entre profissionais já atuantes nas instituições de saúde.

Para os itens de autoeficácia, inexiste um parâmetro do quão verdadeira é a afirmação, já que diz respeito ao que o profissional acredita sobre si mesmo, sendo uma percepção mais subjetiva. Espera-se, no entanto, que as respostas afirmem a veracidade dos itens, representando que se sentem capazes de enfrentar determinadas situações^26^. No presente estudo, o item com maior ganho na autoeficácia foi o item 26: “Informar o paciente e aos familiares sobre os cuidados paliativos na instituição”. Esse achado reforça a importância da divulgação dos CP na atual realidade do hospital, em que o assunto é tão pouco abordado que interferiu no conhecimento da equipe de enfermagem em relação à presença do grupo interdisciplinar de CP na instituição. Dessa forma, evidencia-se a importância da presente pesquisa em preencher uma lacuna no contexto do estudo.

O papel do enfermeiro no cuidado de pacientes com necessidades em CP está associado às diversas práticas essenciais da enfermagem em múltiplas áreas, como o cuidado direto ao paciente e à família, a liderança e o gerenciamento do ambiente, bem como a garantia da segurança do paciente. No entanto, há uma necessidade maior de enfermeiros envolvidos em atividades de pesquisas especificamente voltadas ao tema dos CP, que são essenciais para a prática baseada em evidências. Dessa forma, há uma oportunidade de preencher lacunas por meio de publicações científicas, pois, ao abordar as recomendações relacionadas à pesquisa, à educação e às políticas públicas, as partes interessadas podem trabalhar para fortalecer o papel do enfermeiro em CP e aprimorar os resultados da assistência prestada aos pacientes e seus familiares^27^.

Entende-se como limitação do estudo a ausência de ensino prático associado ao conteúdo teórico durante a intervenção educativa, o que auxiliaria no desenvolvimento com benefícios mais significativos, não apenas no ganho de conhecimento, mas, principalmente, na autoeficácia, contribuindo para que os profissionais se sentissem mais seguros e confiantes na aplicação dos conhecimentos adquiridos em sua prática profissional. Além disso, o tempo destinado para a realização da intervenção educativa foi considerado limitado para a abordagem aprofundada de todos os aspectos relacionados ao tema.

Os referidos resultados apontam um aumento nas respostas assertivas entre o pré-teste e o pós-teste, o que reforça a relevância do estudo e a necessidade de ações educativas no serviço de saúde voltadas à equipe de enfermagem. Isso evidencia que a intervenção educativa é uma importante ferramenta no processo de ensino-aprendizagem sobre essa temática. Ressalta-se que a implementação desses cuidados pode mitigar o sofrimento do paciente e de seus familiares, proporcionar maior conforto e qualidade na assistência, de modo a reduzir a obstinação terapêutica. 

O estudo contribui significativamente para o avanço do conhecimento na área dos CP ao demonstrar a efetividade de uma intervenção educativa. Poderá, portanto, contribuir para o cuidado de enfermagem mais planejado e direcionado quanto às orientações, diálogos e condutas em saúde para essa população específica. No âmbito científico e acadêmico, a pesquisa fortalece a base de evidências sobre intervenções educativas em enfermagem, incentivando novas pesquisas voltadas à humanização do cuidado e à ampliação do acesso aos CP de forma abrangente e eficaz.

Dessa forma, os profissionais de enfermagem devem estar dispostos a aplicar na prática as evidências disponíveis na literatura, visando obter os melhores desfechos em saúde. A prática profissional em CP deve ser sustentada por uma estrutura sólida de conhecimento, ética, princípios, aplicação do método científico e desenvolvimento de um plano de cuidados no processo do pensamento crítico e clínico, com foco na tomada de decisão e na resolução de problemas, interferindo na melhora da qualidade de vida, conforto e dignidade dos pacientes e seus respectivos familiares e cuidadores.

## Conclusão

A intervenção educativa foi eficaz para o ganho do conhecimento e da autoeficácia em CP entre os profissionais de enfermagem, sendo observada uma melhora significativa, principalmente no conhecimento. Para o ganho de conhecimento, o destaque foi para as questões medicamentosas para tratamento da dor. Já quanto à autoeficácia, ressalta-se o maior ganho no item relacionado ao profissional conduzir o paciente e seus familiares aos serviços de CP na própria instituição.

Os resultados podem subsidiar a elaboração e a efetivação de intervenções educativas, especificamente em CP, nos serviços de saúde, a fim de minimizar as lacunas consideradas relevantes nessa abordagem.

## Data Availability

Todos os dados gerados ou analisados durante este estudo estão incluídos neste artigo publicado.

# Translation: Intervención educativa a los profesionales de enfermería sobre cuidados paliativos: un estudio cuasiexperimental*

**DOI:** 10.1590/1518-8345.8026.4821

**Authors:** Joyce Assunção Barros, Taciana Fernandes Araújo Ferreira, Fabiana Bolela, Raquel Pan, Silmara Elaine Malaguti Toffano, Adriana Cristina Nicolussi

**Affiliations:** 1 Universidade Federal do Triângulo Mineiro, Uberaba, MG, Brasil.; 2 Becaria de la Fundação de Amparo à Pesquisa do Estado de Minas Gerais (FAPEMIG), Brasil.; 3 Universidade de São Paulo, Escola de Enfermagem de Ribeirão Preto, Centro Colaborador de la OPS/OMS para el Desarrollo de la Investigación en Enfermería, Ribeirão Preto, SP, Brasil.

**Keywords:** Cuidados Paliativos, Grupo de Enfermería, Educación Continua en Enfermería, Conocimiento, Autoeficacia, Atención Terciaria de Salud

## Abstract

**Objetivo:**

evaluar el conocimiento y la autoeficacia de los profesionales de enfermería sobre los cuidados paliativos antes y después de una intervención educativa.

**Método:**

estudio cuasiexperimental, realizado en un hospital universitario, con profesionales de enfermería que atienden a adultos y ancianos. La recopilación de datos se realizó en dos momentos: en el primero, se aplicó el cuestionario sociodemográfico y el instrumento *Bonn Palliative Care Knowledge Test*, que evalúa los conocimientos y la autoeficacia en cuidados paliativos; inmediatamente después, se llevó a cabo la intervención educativa sobre el tema. En un segundo momento, tras cuatro semanas, se volvió a aplicar el instrumento que evalúa los conocimientos y la autoeficacia. Los datos se evaluaron con el programa *Statistical Package for the Social Sciences*, versión 21.0.

**Resultados:**

participaron 179 profesionales, con una edad media de 40,17 años y predominio del sexo femenino. El puntaje de conocimiento obtuvo medias más altas después de la intervención (preprueba=11,77; posprueba=15,03) mientras que el puntaje de autoeficacia fue en una escala menor (preprueba=3,21; posprueba=3,38), aunque ambos fueron estadísticamente significativos (p<0,01).

**Conclusión:**

la intervención educativa fue efectiva para adquirir conocimiento y autoeficacia en cuidados paliativos en profesionales de enfermería. Estos resultados pueden apoyar el desarrollo y la difusión de prácticas de cuidados paliativos en los servicios de salud.

## Destacados

**(1)** La intervención educativa tuvo un impacto positivo en el conocimiento de los profesionales. **(2)** El número de respuestas correctas después de la intervención educativa aumentó. **(3)** Se debe fomentar el proceso de enseñanza-aprendizaje en cuidados paliativos. **(4)** Los resultados pueden apoyar el desarrollo de otras intervenciones educativas. **(5)** La implementación de esta atención puede proporcionar una atención de mayor calidad.

## Introducción

Los cuidados paliativos (CP) están destinados a las personas con enfermedades o afecciones potencialmente mortales que causan limitaciones y pérdida progresiva de funcionalidad. Sus beneficios y objetivo principal son prevenir y tratar el sufrimiento que el paciente, la familia y el cuidador pueden experimentar, ya sea físico, psicológico, social y/o espiritual^1^.

A pesar de muchos avances en Brasil, los CP aún no están muy extendidos, tanto en la atención en servicios especializados como en el área de educación, ya que existen lagunas en el conocimiento de los profesionales de la salud, falta de capacitación con los equipos que ya trabajan y ausencia de contenido en la matriz curricular de la educación de pregrado^2^.

Un análisis de la comprensión de los profesionales de la salud sobre los CP mostró que tienen poco conocimiento o tienen una visión distorsionada del tema. Tales datos son preocupantes, ya que la falta de capacitación genera inseguridad con relación a cómo actuar en situaciones específicas, privando a los pacientes y familiares de recibir los beneficios que los CP puede proporcionar^3^.

El Ministerio de Salud (MS) brasileño ha estado contribuyendo a la implementación de este enfoque en el país. El 7 de diciembre de 2023 se publicó la Resolución N.º 729, que aprobó la Política Nacional de Cuidados Paliativos (PNCP) en el Sistema Único de Salud (SUS), que prevé la garantía de acceso a esta asistencia en cualquier punto de la Red de Atención a la Salud (RAS). Ante este escenario, se entiende que la demanda relacionada con la atención de estos pacientes no será atendida solo por equipos especializados en CP, ya que no hay números suficientes en Brasil. Así, los profesionales no especializados, que ya actúan en la RAS, serían responsables de atender la mayoría de las demandas relacionadas con los CP^4^.

Sin embargo, para que esto suceda, estos profesionales necesitan desarrollar conocimientos y habilidades sobre el tema, para aplicar esta práctica correctamente, siguiendo los principios y ofreciendo una atención de calidad, sin dejar a la población desatendida. Los profesionales de enfermería son responsables de atender la mayoría de las demandas; así, las acciones educativas son necesarias como parte de la planificación del sistema de salud, de modo que no solo se favorezca el acceso sino también la resolución de las necesidades de salud de los usuarios que necesitan este cuidado^5^.

La educación en servicio es un proceso continuo y dinámico para la construcción del conocimiento profesional. Las actividades educativas proporcionan pensamiento crítico y transformación de la realidad en la que se inserta el profesional. Además, las prácticas de enfermería siempre están asociadas a acciones educativas, promoviendo cambios en sus actividades, asegurando la calidad del cuidado y la mejora profesional continua^6^.

Así, en vista del bajo desarrollo de los CP en Brasil, posiblemente asociado a lagunas en el conocimiento de los profesionales y considerando que es una práctica esencial en los sistemas de salud, especialmente en este contexto de transición sociodemográfica, con una creciente demanda de pacientes que puedan beneficiarse de este enfoque, se vuelve relevante desarrollar acciones destinadas a construir conocimiento y sensibilizar sobre el tema, con el objetivo de calificar la atención prestada a la población^5^.

En este contexto, la identificación de lo que el profesional tiene de conocimiento debe ser considerada en la práctica docente, ya que facilita el proceso de aprendizaje y su transmutación en acciones prácticas para cambiar la realidad^7^. Ante el escenario anterior, este estudio tuvo como objetivo evaluar el conocimiento y la autoeficacia de los profesionales de enfermería sobre los CP antes y después de una intervención educativa.

## Método

### Diseño del estudio

Se trata de un estudio cuasiexperimental, del tipo antes y después, en el que se evaluaron los mismos sujetos en el preprueba y posprueba de una intervención educativa, que siguió la lista de verificación *Template for Intervention Description and Replication* (TIDieR).

### Local

La investigación se llevó a cabo en un hospital público de enseñanza de la ciudad de Uberaba, en el estado de Minas Gerais (MG), Brasil. El hospital tiene 302 camas activas y atiende la demanda del SUS en una macrorregión de Minas Gerais. La institución es una referencia para mediana y alta complejidad^8^.

### Periodo

La recolección de datos se realizó de mayo a septiembre de 2024.

### Muestra y criterios de inclusión

La población de estudio estuvo compuesta por profesionales de enfermería (enfermeros, técnicos de enfermería y auxiliares de enfermería) con edad igual o superior a 18 años, que actuaban en el cuidado directo de pacientes adultos/ancianos, de los siguientes sectores en régimen de hospitalización: ortopedia, medicina interna, clínica quirúrgica, unidad de enfermedades infecciosas y parasitarias, neurología, ginecología y obstetricia, oncohematología, unidad de cuidados intensivos I, II y urgencias coronarias y de adultos. Se excluyeron los profesionales que estaban de vacaciones o fuera del trabajo durante el período de estudio.

El tamaño de la muestra se calculó después de analizar el estudio piloto con 18 profesionales. El nivel de significancia considerado fue un error tipo I (α=0,01), un error tipo II (β=0,1) y un poder estadístico del 90%. Considerando el conocimiento de CP como resultado primario, el aumento medio de los puntajes de la prueba previa a la prueba posterior fue de 5,72 puntos (DE=3,10), lo que equivale a una ganancia de más del 50%. Con base en estos valores, el tamaño de la muestra se realizó utilizando el programa *Power Analysis and Sample Size* (PASS), resultando en un total de 30 participantes. Cabe señalar que los participantes en el estudio piloto no formaron parte del estudio principal.

Dado que la magnitud del efecto de la intervención en el estudio piloto fue mayor de lo esperado, se decidió, como precaución, que el estudio principal debería incluir un número mayor de participantes que el recomendado en el estudio piloto. Como la intervención se realizó en asociación y registrada en el Servicio de Educación de Enfermería (SEE) del mencionado hospital, se decidió invitar a todos los profesionales de enfermería interesados en participar del estudio. Por lo tanto, se utilizó un muestreo no probabilístico por conveniencia.

Así, 214 profesionales de enfermería participaron en el estudio preprueba; sin embargo, en la etapa de aplicación posterior a la prueba, hubo una pérdida de 35 participantes, con la muestra final de 179 profesionales.

### Instrumentos de recolección de datos

Para caracterizar la muestra se utilizó un cuestionario sociodemográfico y profesional elaborado por los propios autores, que contenía las siguientes variables: edad, sexo, nivel de educación, categoría profesional, título, especialización (posgrado), sector de empleo, tiempo total de experiencia profesional y turno de trabajo.

Para evaluar el conocimiento y la autoeficacia en CP, se utilizó el instrumento *Bonn Palliative Care Knowledge Test* (BPW), desarrollado en Alemania en 2011^9^, traducido y adaptado culturalmente al contexto brasileño en 2018^10^. Contiene 38 preguntas, 23 de las cuales están destinadas a evaluar el conocimiento, con temas sobre el control del dolor y otros síntomas, el conocimiento general sobre el tema y las actitudes hacia la muerte y el morir. De acuerdo con las instrucciones del instrumento, los ítems de conocimiento numerados del 1 al 4, del 6 al 10, del 12, del 14 y del 16 al 20 deben considerarse incorrectos, mientras que los ítems 5, 11, 13, 15, 21 al 23 se consideran correctos.

Las 15 preguntas restantes evalúan la autoeficacia en la administración de CP y proporcionan información sobre la confianza de los participantes para implementar lo aprendido en la práctica clínica. Las preguntas están organizadas en el formato de una escala Likert, cuyas respuestas incluyen “correcto”, “más correcto que incorrecto”, “más incorrecto que correcto” o “incorrecto”^9^ ^-^ ^10^.

Para la escala de conocimiento, la puntuación general representa la suma de los ítems considerados correctos y puede variar de cero (si el encuestado se equivocó en todos los ítems) a 23 (si acertó en todos los ítems). Cuanto mayor sea la puntuación, mayor será el conocimiento del profesional. En la escala de autoeficacia, el puntaje global representa la media de las respuestas a los ítems de la escala y puede variar de cero (se siente incapaz) a cuatro (se siente totalmente capaz), es decir, cuanto más cerca de cuatro, más capaz se siente el profesional de realizar una determinada acción descrita en el instrumento^9^ ^-^ ^10^.

### Etapa operacional

El contenido programático de la clase de intervención educativa se desarrolló basándose en el manual de CP del Ministerio de Salud en colaboración con el Hospital Sírio-Libanês (2023)^1^ y en la bibliografía vigente, centrándose en cuestiones importantes para el conocimiento en PC.

Así, los temas de abordaje seleccionados y elaborados fueron: a) concepto actualizado y principios de CP; b) la importancia del tema para los profesionales de la salud, con la presentación de datos epidemiológicos; c) la relación entre CP y el tratamiento modificador de la enfermedad, destacando que los objetivos de la terapia paliativa estarán de acuerdo con la evolución funcional y clínica de cada paciente; d) la importancia de la atención multiprofesional; e) identificación y control de los síntomas físicos prevalentes en esta demanda (dolor, náuseas y vómitos, estreñimiento y disnea); f) los principales síntomas psíquicos (ansiedad, depresión y delirio); g) los aspectos sociales más comunes, como la pérdida de identidad social, la pérdida del rol social y familiar y el aislamiento social; h) espiritualidad; i) cuidados al final de la vida, destacando que los tratamientos propuestos deben ser evaluados en relación con su proporcionalidad frente al proceso de finitud; j) comunicación efectiva entre el equipo, el paciente y la familia; k) los servicios que están disponibles en el hospital para satisfacer la demanda de CP y cómo solicitar este servicio.

Después de la preparación de la clase, el contenido fue enviado para su validación, que ocurrió de forma independiente, a través de una carta de invitación, vía electrónica (correo electrónico), a 13 doctores previamente seleccionados a través del análisis del Currículo Lattes, que tenían conocimiento sobre el constructo en cuestión. De los 13 doctores, siete no respondieron a ninguno de los contactos electrónicos y tres se negaron a participar. Así, tres doctores formaron parte de la validación de contenido.

Para quienes aceptaron participar en la validación, se envió un nuevo correo electrónico que contenía el Formulario de Consentimiento Informado (FCI) y el Instrumento para la Validación de Contenidos Educativos en Salud (IVCES). El plazo solicitado para la devolución del instrumento por parte del especialista a los investigadores fue de 10 días, y este plazo puede flexibilizarse, si es necesario.

El IVCES tiene 18 ítems, y se divide en tres grupos: objetivos (1-5), estructura y presentación (6-15) y relevancia (16-18), con opciones de respuesta utilizando la escala *delirium*, donde 0 = en desacuerdo, 1 = parcialmente de acuerdo y 2 = totalmente de acuerdo. Además de la partitura del instrumento, el especialista también podía registrar sus consideraciones en texto plano, si era necesario^11^.

Tras la finalización y devolución del instrumento por parte de todos los expertos, se evaluó el acuerdo entre los jueces respecto a la validación del contenido. Para ello, se calculó el Índice de Validez de Contenido (IVC), que corresponde a la división entre la suma del número de respuestas de acuerdo (ítems marcados como 2) por el número total de respuestas, considerando como aceptable el valor mínimo recomendado del 80%.

El experto 1 otorgó la puntuación máxima (100 %), marcando la opción ^“^totalmente de acuerdo” en los 18 ítems del instrumento. Por su parte, los expertos 2 y 3 obtuvieron una puntuación del 80%, con 15 ítems evaluados como ^“^totalmente de acuerdo^”^ y 3 como ^“^parcialmente de acuerdo^”^.

Se destaca que este proceso de validación es importante, ya que evalúa la claridad y comprensión del público objetivo, su representatividad al abordar adecuadamente el universo al que se propone y, también, busca evitar la inclusión de elementos innecesarios^11^.

Después de la validación del contenido, la intervención educativa fue registrada en el SEE del mencionado hospital. Se realizaron reuniones con los investigadores, el enfermero del SEE y los Responsables Técnicos (RT) de cada sector participante en la investigación, con el objetivo de definir y programar las fechas y horarios de las clases. Los RT fueron responsables de difundir y dirigir al equipo de enfermería para participar en la intervención.

A continuación, se probó la intervención educativa en un estudio piloto con 18 profesionales y, luego, se realizaron ajustes solo en relación con la estructura de la presentación, sin cambiar el contenido, con el fin de optimizar el tiempo de intervención en el horario programado. Después de que se llevó a cabo el piloto, comenzó la fase de intervención educativa del estudio principal.

### Recolección de datos

La intervención educativa se realizó a través de una presentación expositiva con el apoyo del recurso visual *Microsoft Power Point* ^®^ y también utilizó la metodología participativa y dialógica, con el objetivo de permitir el intercambio de experiencias, aclaración de dudas y discusión de las preguntas planteadas por los participantes sobre el tema.

La recolección de datos se realizó en dos momentos (preprueba y posprueba). El primero, colectivamente y en pequeños grupos con los equipos de enfermería, en un aula reservada en el propio hospital, con programación previa de fechas y horarios dentro del horario de trabajo de los profesionales, en los tres turnos (mañana, tarde y noche). Se necesitaron varias disponibilidades de horarios y días de clases debido a la imposibilidad de que los profesionales abandonaran cada sector al mismo tiempo, y en total, se ofrecieron 51 clases.

En cada reunión se presentaron los objetivos de la investigación, así como el FCI. Después de la aceptación y firma, se aplicó el cuestionario sociodemográfico y profesional y el instrumento BPW. Se estableció un tiempo aproximado de veinte minutos para la finalización de los instrumentos, los cuales fueron llenados individualmente. Este tiempo está de acuerdo con las recomendaciones de los autores del estudio original del instrumento y también se determinó en base al estudio piloto. A continuación, se realizó la educación en los servicios de salud en CP, realizada por los propios investigadores. La clase duró aproximadamente cuarenta minutos, totalizando aproximadamente una hora para toda la actividad.

En el segundo momento de recolección de datos, cuatro semanas después de la intervención educativa, solo se volvió a aplicar el instrumento BPW. Esta etapa se dio de manera individual, a través de una búsqueda activa de participantes en los sectores en los que trabajaban. La finalización del instrumento duró aproximadamente diez minutos.

### Tratamiento y análisis de datos

Los datos recolectados fueron ingresados en una hoja de cálculo de Excel^®^ del programa de Windows^®^ mediante la técnica de doble tipado, para su posterior validación. La identificación de cada participante del estudio se realizó a través de códigos. A continuación, la base de datos validada fue importada al *Statistical Package for the Social Sciences* (SPSS) versión 21.0 para su procesamiento y análisis^12^.

Los datos se analizaron mediante estadística descriptiva con medidas de tendencia central (media) y dispersión (desviación estándar) para las variables cuantitativas y frecuencias absolutas y relativas para las variables categóricas. Se evaluó la diferencia entre las medias previas y posteriores a la prueba, tanto en cuanto al conocimiento como a la autoeficacia en CP. Se han verificado debidamente todos los requisitos para el uso de pruebas paramétricas, incluida la normalidad de los datos y el análisis de valores atípicos. Por lo tanto, se aplicó la prueba T pareada, nivel de significancia I α=0,01, considerando un intervalo de confianza del 99%.

Para verificar la magnitud del efecto de la intervención, se utilizó una aplicación disponible en el sitio *web* de la Psychometrica.de (https://www.psychometrica.de/effect_size.html)^13^. La interpretación de los tamaños del efecto siguió la siguiente clasificación de Cohen: d de Cohen (0,0 a 0,1) = sin efecto; d de Cohen (0,2 a 0,4) = efecto pequeño; d de Cohen (0,5 a 0,7) = efecto moderado y d de Cohen (0,8 a ≥1,0) = efecto grande.

### Aspectos éticos

La investigación fue registrada en la *Rede Pesquisa* (Red de Investigación), herramienta que forma parte de la Red de la Empresa Brasileña de Servicios Hospitalarios (EBSERH), bajo el código n.º 3738 y obtuvo el consentimiento de la Dirección de Docencia e Investigación del mencionado hospital y fue aprobada por el Comité de Ética en Investigación de la institución proponente, dictamen n.º 6.510.776, respetando los principios éticos.

## Resultados

De los 179 participantes evaluados en este estudio, la mayoría (82,1%) eran mujeres, con una edad media de 40,17 años (DE = 8,5), con una edad mínima de 23 años y una máxima de 64 años.

En cuanto a los datos profesionales, la mayoría (69,3%) estuvo compuesta por técnicos de enfermería, con predominio de los que trabajaban en el sector de clínica médica (26,3%) y turno de noche (36,3%), con más de 10 años de experiencia profesional (64,2%). Con respecto a los estudios de posgrado, 44,1% relataron haber realizado alguna especialización, predominando cursos en las áreas de cuidados intensivos (30,37%) y urgencia y emergencia (26,58%). Solo un participante relató tener un posgrado en el área de CP.

En cuanto a la puntuación de conocimiento y autoeficacia en CP, hubo un aumento en el número de respuestas correctas después de la intervención educativa, como se muestra en la Tabla 1.

**Tabla 1:** Distribución de los puntajes de la muestra medidos por la *Bonn Palliative Care Knowledge Test* (n = 179). Uberaba, MG, Brasil, 2024

Variables	Promedio	De*	Mínimo	Máximo
Puntuación de conocimientos previa a la prueba	11,77	2,56	4	18
Puntuación de conocimientos posterior a la prueba	15,03	3,64	8	23
Puntuación de autoeficacia previa a la prueba	3,21	0,48	1,73	4
Puntuación de autoeficacia posterior a la prueba	3,38	0,43	2	4

^*^ De = Desviación estándar

En cuanto a los constructos de conocimiento, los promedios aumentaron considerablemente después de la educación en servicio, con una ganancia de tres puntos en el desempeño promedio. Sin embargo, los resultados relacionados con la autoeficacia aumentaron a menor escala, con una ganancia de solo 0,1. Ambos fueron considerados estadísticamente significativos (p<0,01). En cuanto al efecto de la magnitud de la intervención, la prueba de conocimiento se consideró de gran efecto (0,8 a 1,0) y la prueba de autoeficacia tuvo un efecto moderado (0,5 a 0,7), como se muestra en la Tabla 2. Cabe destacar que, para las evaluaciones del instrumento, cuanto más cerca de 23, mayor es el conocimiento y cuanto más cerca de cuatro, mayor es la autoeficacia percibida.

**Tabla 2:** Comparación de la diferencia entre las medias previas y posteriores a la prueba del equipo de enfermería del *Bonn Palliative Care Knowledge Test* (n = 179). Uberaba, MG, Brasil, 2024

	Prueba de muestras pareadas			
	Promedio	De*	*p* ^†^	d^‡^
Puntuación de conocimientos previos y posteriores a la prueba	-3,26	4,10	0,000	0,83
Puntuación de autoeficacia previa y posterior a la prueba	-0,17	0,49	0,000	0,52

*De = Desviación estándar; ^†^Valor de p = Significativo <0,001; ^‡^d = d de Cohen

La Tabla 3 presenta los resultados de la prueba previa y posterior de cada ítem de la evaluación del conocimiento de BPW y la Tabla 4 de la evaluación de autoeficacia. La pregunta que mostró mayor ganancia en respuestas correctas después de la intervención fue el ítem dos (diferencia porcentual de 35,2%), que se refiere al uso de antiinflamatorios con el uso de opioides. En cuanto a los datos relativos a la autoeficacia, se observó una menor variación entre la prueba previa y la prueba posterior, ya que la escala solo varía de uno a cuatro y cabe destacar que, en la prueba previa, los resultados se acercaron al valor límite. Entre los ítems que tuvieron mayor ganancia después de la educación en servicio, se destacó el ítem 26 (diferencia porcentual de 17,2%), que aborda la información sobre CP en la propia institución.

**Tabla 3:** Evaluación de cada elemento del conocimiento previo y posterior a la prueba del equipo de enfermería del instrumento *Bonn Palliative Care Knowledge Test* (n = 179). Uberaba, MG, Brasil, 2024.

Preguntas	Respuestas correctas		
	Preprueba	Postprueba	
Parte 1 - Conocimiento	n (%)*	n (%)*	Dif (%)^†^
1. Los cuidados paliativos nunca deben combinarse con tratamientos curativos.	120 (67,0)	161 (89,9)	22,9
2. Los medicamentos antinflamatorios no esteroides no deben ser usarse durante el uso regular de opioides.	91 (50,8)	154 (86,0)	35,2
3. La administración subcutánea de líquidos (hipodermoclisis) es necesaria para aliviar la sequedad bucal (xerostomía) en pacientes en fase final de la vida.	97 (54,2)	132 (73,7)	19,7
4. El uso de parches transdérmicos para el control del dolor inestable es apropiado en pacientes en la fase final de la vida.	55 (30,7)	102 (57,0)	26,3
5. Las terapias complementarias (p. ej., fisioterapia) son importantes para el manejo del dolor.	142 (79,3)	156 (87,2)	7,9
6. Para los miembros de la familia, siempre es importante permanecer al lado del paciente moribundo.	13 (7,3)	48 (26,8)	18,9
7. El estreñimiento debe aceptarse como un síntoma secundario, ya que la reducción del dolor es más importante.	101 (56,4)	102 (57,0)	0,6
8. Los cuidados paliativos requieren una cercanía emocional constante.	32 (17,9)	46 (25,7)	7,8
9. Las personas con edad avanzada han aprendido de diversas experiencias de pérdida a sobrellevar el duelo por sí mismas.	112 (62,6)	127 (70,9)	8,3
10. La filosofía de los Cuidados Paliativos implica no realizar intervenciones para prolongar la vida.	62 (34,6)	91 (50,8)	16,2
11. El umbral del dolor se reduce por la ansiedad o la fatiga.	59 (33,0)	84 (46,9)	13,9
12. Los pacientes con enfermedades potencialmente mortales siempre deben saber la verdad para que puedan prepararse para la muerte.	33 (18,4)	59 (33,0)	14,6
13. Los miembros del equipo no necesitan tener una creencia o religión en particular para brindar atención espiritual a los pacientes en las etapas finales de la vida.	140 (78,2)	171 (95,5)	17,3
14. El paciente que recibe Cuidados Paliativos debe aceptar la muerte.	125 (69,8)	130 (72,6)	2,8
15. Las habilidades de comunicación se pueden aprender.	177 (98,9)	179(100,0)	1,1
16. La muerte de un paciente no debe ser reportada a otros pacientes cercanos a él y en una situación similar para evitar disturbios.	37 (20,7)	56 (31,3)	10,6
17. Los aspectos médicos del tratamiento siempre tienen prioridad en los Cuidados Paliativos.	115 (64,2)	153 (85,5)	21,3
18. Cuando un paciente muere, se deben evitar las ceremonias visibles y los ritos ostentosos para evitar la inquietud en otros pacientes.	57 (31,8)	77 (43,0)	11,2
19. No se recomienda el uso de antidepresivos para el tratamiento del dolor.	142 (79,3)	174 (97,2)	17,9
20. No se requieren analgésicos adyuvantes cuando el paciente recibe opioides.	138 (77,1)	165 (92,2)	15,1
21. La fase final comprende los últimos tres días de vida.	39 (21,8)	68 (38,0)	16,2
22. Los sentimientos personales del cuidador (p. ej., aversión) pueden aparecer durante la atención del paciente	106 (59,2)	122 (68,2)	9,0
23. Las necesidades fisiológicas (por ejemplo, la sexualidad) siguen siendo importantes en la fase final de la vida.	115 (64,2)	135 (75,4)	11,2

*n (%) = Número (porcentaje); ^†^Dif (%) = Diferencia porcentual

**Tabla 4:** Evaluación de cada ítem de autoeficacia preprueba y posprueba del equipo de enfermería del instrumento *Bonn Palliative Care Knowledge Test* (n = 179). Uberaba, MG, Brasil, 2024

Parte 2 - Autoeficacia			
Creo que soy capaz de...	Prepuebla n (%)*	Pospuebla n (%)*	Dif (%)^†^
24. Recopilar información que describa la intensidad del dolor del paciente de manera objetiva.	164 (91,6)	175 (99,5)	7,9
25. Aconsejar al paciente sobre cómo aliviar las náuseas.	153 (85,5)	176 (98,4)	12,9
26. Informar al paciente y a sus familiares sobre los cuidados paliativos en la institución.	122 (68,2)	153 (85,4)	17,2
27. Convencer al médico de cabecera sobre las necesidades de los cuidados paliativos.	75 (41,9)	94 (52,6)	10,7
28. Identificar y discutir problemas concretos en el contexto social del paciente.	126 (70,4)	140 (78,2)	7,8
29. Facilitar el contacto con una unidad.	105 (58,7)	122 (68,2)	9,5
30. Hablar con un paciente ansioso y sus familiares para que se sientan seguros.	165 (92,1)	166 (92,7)	0,6
31. Identificar las múltiples necesidades de un paciente al final de la vida y reaccionar adecuadamente.	162 (88,9)	162 (88,9)	0,0
32. Ofrecer alternativas de relajación adecuadas a un paciente con dolor.	157 (87,7)	158 (88,2)	0,5
33. Hablar con un paciente que expresa el deseo de acelerar la muerte.	123 (68,7)	134 (74,8)	6,1
34. Realizar una higiene bucal adecuada de los pacientes al final de la vida.	178 (99,4)	179 (100,0)	0,6
35. Educar al paciente sobre los posibles efectos secundarios de los medicamentos recetados.	169 (94,4)	175 (97,8)	3,4
36. Identificar problemas psicológicos específicos de los pacientes.	134 (74,9)	157 (87,7)	12,8
37. Integrar aspectos culturales relacionados con la muerte y el morir en el cuidado de pacientes al final de la vida.	119 (66,5)	143 (79,9)	13,4
38. Empatizar con las necesidades de los pacientes y sus familias en diferentes situaciones de la vida y reaccionar adecuadamente ante ellas.	174 (97,3)	176 (98,3)	1,0

*n (%) = Número (porcentaje); ^†^Dif (%) = Diferencia porcentual

## Discusión

El análisis de los datos recolectados indicó el predominio del curso de posgrado en cuidados intensivos y urgencia y emergencia. Tal enfoque en la curación y tratamiento de la enfermedad puede influir en el equipo de enfermería para no comprender la importancia de la integración precoz y los beneficios de un abordaje paliativo combinado con terapia modificadora de la enfermedad^1^. 

Las competencias en CP son esenciales para que los profesionales practiquen la alta calidad. Es necesario que comprendan que, tan fundamental como la realización de procedimientos técnicos, es desarrollar habilidades como la evaluación y el control de los síntomas, la comunicación adecuada y la comprensión de la proporcionalidad de la atención, que cambia de acuerdo con la condición clínica y la capacidad de recuperación del paciente^14^.

Estas habilidades son cada vez más importantes en un escenario de cambio epidemiológico, en el que la demanda de CP está aumentando^2^. Los estudios sobre intervenciones educativas contribuyen significativamente a fortalecer la evidencia científica sobre sus beneficios y destacan la relevancia de estas propuestas para mejorar el conocimiento. Además, pueden favorecer cambios de actitudes, modificación de creencias y reducción de barreras en los servicios de salud, resultando en una mejor experiencia del paciente^15^.

En este estudio, la intervención educativa en CP demostró ser efectiva para la adquisición de conocimientos entre los profesionales de enfermería. Un resultado similar se observó en un estudio realizado con 102 enfermeros, que demostró que la educación en servicio contribuye a mejorar la competencia de los profesionales en la prestación de servicios de CP calificados. Sin embargo, se descubrió que el conocimiento básico sobre PC aún es limitado. En este sentido, las intervenciones de aprendizaje, como la educación continua en enfermería, pueden promover avances significativos en la comprensión de los CP y ayudar a superar las brechas relacionadas con el conocimiento y las habilidades profesionales^16^.

Se cree que la modificación del comportamiento de los profesionales ocurre cuando las necesidades internas y externas se transforman en motivación y propósito, mediado por la autoconciencia y la comprensión. La educación en servicios de salud promueve la reflexión crítica sobre los procesos de trabajo, favoreciendo la problematización de la realidad de los servicios y la construcción de soluciones, integrando capacitación, gestión y cuidado para mejorar las prácticas, fortalecer el desempeño del equipo y mejorar la calidad de la atención a la comunidad^17^. Así, se señala que la inserción del proceso educativo en los servicios de salud es fundamental para promover cambios efectivos en la práctica profesional.

Se encontró que, en este estudio, el valor medio para el constructo de conocimiento en CP en la preprueba fue bajo; En cuanto a la autoeficacia, los profesionales respondieron que se sentían capaces de brindar este tipo de atención. Estudios similares nacionales e internacionales corroboran estos resultados^18^ ^-^ ^19^. Sin embargo, no es sorprendente que las personas se consideren competentes incluso con grandes lagunas en el conocimiento, ya que existe una gran diferencia entre poseer conocimiento y poder usarlo bien en diversas circunstancias. La autoeficacia influye en el rendimiento; así, una persona puede considerarse competente debido a la retroalimentación recibida, que puede no estar directamente relacionada con su nivel de conocimiento^20^.

En cuanto a la diferencia en el puntaje de autoeficacia entre la preprueba y la posprueba, se observó una baja ganancia. Este resultado puede estar relacionado con la pequeña varianza de amplitud de la escala (uno a cuatro) y la falta de una metodología que involucre una parte práctica en el tema abordado. Así, para tener un impacto más positivo en la autoeficacia en CP, es importante utilizar diferentes métodos de aprendizaje activo, como la simulación de un caso clínico. Esta estrategia permite a los participantes aprender a evaluar correctamente al paciente, identificar sus necesidades y diseñar un plan de atención. Como resultado, los profesionales tienden a sentirse más seguros de sí mismos en el desempeño de las tareas y demandas asignadas^21^.

Al analizar por separado los ítems del instrumento en el constructo de conocimiento, es importante destacar que las preguntas relacionadas con el uso de medicamentos fueron los ítems que obtuvieron una ganancia significativa en las respuestas correctas en la posprueba (ítems 2, 4 y 19). Por tratarse de preguntas que involucran más conocimientos técnicos, se considera que estos ítems han constituido un desafío en la preprueba para el equipo de enfermería que respondió el cuestionario.

En este sentido, se destaca que contar con conocimientos técnicos, habilidades y sentirse capaz de brindar cuidados con un enfoque paliativo, especialmente en el control del dolor, es esencial para los profesionales de enfermería. El tratamiento del dolor en cuidados paliativos implica una evaluación integral, que tiene en cuenta factores físicos, psicológicos, sociales y espirituales y combina enfoques farmacológicos y no farmacológicos. El equipo de enfermería debe realizar una evaluación exhaustiva, incorporando comportamientos del paciente e información subjetiva y objetiva, con el fin de comunicar adecuadamente los niveles de dolor al equipo interdisciplinario y diseñar un plan de manejo efectivo. Sin embargo, barreras como la falta de conocimiento sobre la evaluación y el manejo del dolor, la mala comunicación entre los profesionales y la ausencia de directrices estandarizadas pueden comprometer el reconocimiento adecuado de la percepción del paciente, lo que hace esencial la preparación técnica y la práctica reflexiva del equipo de salud^22^.

Se encontró que, en este estudio, el ítem 1 también presentó un mayor número de respuestas correctas en la posprueba, que se refiere a la integración de la CP con el tratamiento curativo. Se cree que, en la preprueba, hubo confusión debido a la falta de comprensión de los profesionales sobre el concepto actualizado de CP. Este resultado es de gran importancia para la práctica clínica, ya que la integración temprana de este enfoque promueve la planificación anticipada de la atención, abordando cuestiones como el control del dolor, el manejo de los síntomas físicos y el apoyo psicosocial y espiritual, lo que se refleja en el éxito terapéutico del paciente^23^.

Un estudio realizado en los Países Bajos identificó que, cuando la integración de los CP ocurre tarde, ya durante la fase de final de la vida, los pacientes son hospitalizados con síntomas graves y difíciles de controlar, debido a la falta de reconocimiento oportuno de las necesidades^24^. Este retraso en la elegibilidad se debe a varias barreras, como los estigmas relacionados a la derivación debido a un pronóstico incierto, la dificultad para conocer los criterios para identificar a los pacientes con mayor probabilidad de beneficiarse del seguimiento precoz y la falta de conocimiento de los profesionales sobre el tema^24^ ^-^ ^25^. Estos hallazgos resaltan la importancia del conocimiento y la necesidad de discusión sobre CP, tanto en la academia como entre los profesionales que ya trabajan en instituciones de salud.

Para los ítems de autoeficacia, no existe un parámetro de cuán verdadero es el enunciado, ya que se refiere a lo que el profesional cree sobre sí mismo, siendo una percepción más subjetiva. Se espera, sin embargo, que las respuestas afirmen la veracidad de los ítems, representando que se sienten capaces de enfrentar ciertas situaciones^26^. En el presente estudio, el ítem con mayor ganancia en autoeficacia fue el ítem 26: “Informar al paciente y a sus familiares sobre los cuidados paliativos en la institución”. Este hallazgo refuerza la importancia de la difusión de los CP en la realidad actual del hospital, en la que el tema es tan poco abordado que interfirió con el conocimiento del equipo de enfermería con relación a la presencia del grupo interdisciplinario de CP en la institución. Por lo tanto, se evidencia la importancia de la presente investigación para llenar un vacío en el contexto del estudio.

El papel del enfermero en el cuidado de pacientes con necesidades en CP asociado a varias prácticas de enfermería esenciales en múltiples áreas, como el cuidado directo del paciente y la familia, el liderazgo y la gestión del entorno, así como la garantía de la seguridad del paciente. Sin embargo, existe una mayor necesidad de enfermeros involucrados en actividades de investigación específicamente enfocadas en el tema de CP, que son esenciales para la práctica basada en evidencias. Así, existe la oportunidad de llenar vacíos a través de publicaciones científicas, ya que, al abordar recomendaciones relacionadas con la investigación, la educación y las políticas públicas, los actores pueden trabajar para fortalecer el papel de los enfermeros de CP y mejorar los resultados de la atención prestada a los pacientes y sus familias^27^.

La limitación del estudio es la ausencia de enseñanza práctica asociada al contenido teórico durante la intervención educativa, lo que ayudaría en el desarrollo con beneficios más significativos, no solo en la ganancia de conocimientos, sino principalmente en la autoeficacia, contribuyendo a que los profesionales se sientan más seguros y confiados en la aplicación de los conocimientos adquiridos en su práctica profesional. Además, el tiempo destinado a realizar la intervención educativa se consideró limitado para el abordaje en profundidad de todos los aspectos relacionados con el tema.

Estos resultados indican un aumento de las respuestas asertivas entre la preprueba y la posprueba, lo que refuerza la relevancia del estudio y la necesidad de acciones educativas en el servicio de salud dirigidas al equipo de enfermería. Esto demuestra que la intervención educativa es una herramienta importante en el proceso de enseñanza-aprendizaje sobre este tema. Se enfatiza que la implementación de esta atención puede mitigar el sufrimiento de los pacientes y sus familias, brindar mayor comodidad y calidad de atención, con el fin de reducir la obstinación terapéutica.

El estudio contribuye significativamente al avance del conocimiento en el área de CP al demostrar la efectividad de una intervención educativa. Por lo tanto, puede contribuir para una atención de enfermería más planificada y dirigida en términos de orientaciones, diálogos y conductas de salud para esta población específica. En los ámbitos científico y académico, la investigación fortalece la base de evidencia sobre intervenciones educativas en enfermería, incentivando nuevas investigaciones dirigidas a la humanización del cuidado y la ampliación del acceso a los CP de forma integral y efectiva.

Por lo tanto, los profesionales de enfermería deben estar dispuestos a aplicar la evidencia disponible en la literatura en la práctica, con el objetivo de obtener los mejores resultados de salud. La práctica profesional en CP debe estar respaldada por una sólida estructura de conocimientos, ética, principios, aplicación del método científico y desarrollo de un plan de cuidados en el proceso de pensamiento crítico y clínico, enfocándose en la toma de decisiones y resolución de problemas, interfiriendo en la mejora de la calidad de vida, comodidad y dignidad de los pacientes y sus respectivas familias y cuidadores.

## Conclusión

La intervención educativa fue efectiva para la adquisición de conocimiento y autoeficacia en CP entre los profesionales de enfermería, observándose una mejora significativa, especialmente en el conocimiento. Para adquirir conocimiento, lo más destacado fueron los problemas de medicación para el tratamiento del dolor. En cuanto a la autoeficacia, se destaca la mayor ganancia en el ítem relacionado con el profesional que lleva al paciente y a su familia a los servicios de CP en la propia institución.

Estos resultados pueden apoyar el desarrollo e implementación de intervenciones educativas, específicamente en CP, en los servicios de salud, con el fin de minimizar las brechas consideradas relevantes en este enfoque.

## Data Availability

Todos los datos generados o analizados durante este estudio están incluidos en este artículo publicado.
